# Genome Skimming Illuminates Hidden Species Diversity and Symbiodiniaceae Associations in East Pacific *Pocillopora* Corals

**DOI:** 10.1093/gbe/evaf235

**Published:** 2025-12-03

**Authors:** Michael T Connelly, Victoria Marie Glynn, Anabell Cornejo, Tatiana Villalobos-Cubero, Joan Kleypas, Juan José Alvarado, Margarita Brandt, Cheryl A Logan, Ana M Palacio-Castro, Jean-François Flot, Matthieu Leray, Sean R Connolly, Andrea M Quattrini

**Affiliations:** Department of Invertebrate Zoology, Smithsonian Institution National Museum of Natural History, Washington, DC, USA; Smithsonian Tropical Research Institute, Naos Marine Laboratories, Ancón, Panamá, Republic of Panamá; Department of Biology, University of Vermont, Burlington, VT 05405, USA; Smithsonian Tropical Research Institute, Naos Marine Laboratories, Ancón, Panamá, Republic of Panamá; Raising Coral Costa Rica (RCCR), San José, Costa Rica; Raising Coral Costa Rica (RCCR), San José, Costa Rica; Escuela de Biología, Universidad de Costa Rica, San José, Costa Rica; Centro de Investigación en Biodiversidad y Ecología Tropical (CIBET), Universidad de Costa Rica, San José, Costa Rica; Centro de Investigación en Ciencias del Mar y Limnología (CIMAR), Universidad de Costa Rica, San José, Costa Rica; Colegio de Ciencias Biológicas y Ambientales, Universidad San Francisco de Quito USFQ, Quito, Ecuador; Galápagos Science Center GSC, Universidad San Francisco de Quito USFQ & University of North Carolina at Chapel Hill UNC, Puerto Baquerizo Moreno, Galápagos, Ecuador; Department of Marine Science, California State University, Monterey Bay, CA, USA; Cooperative Institute for Marine and Atmospheric Studies (CIMAS), University of Miami, Miami, FL, USA; Atlantic Oceanographic and Meteorological Laboratory, National Oceanic and Atmospheric Administration, Miami, FL, USA; Department of Evolutionary Biology & Ecology, Université Libre de Bruxelles (ULB), Brussels, Belgium; Interuniversity Institute of Bioinformatics in Brussels, Brussels, Belgium; Brussels Laboratory of the Universe (BLU), Brussels, Belgium; Smithsonian Tropical Research Institute, Naos Marine Laboratories, Ancón, Panamá, Republic of Panamá; Smithsonian Tropical Research Institute, Naos Marine Laboratories, Ancón, Panamá, Republic of Panamá; Department of Invertebrate Zoology, Smithsonian Institution National Museum of Natural History, Washington, DC, USA

**Keywords:** coral, genomics, evolution, species delimitation, symbiodiniaceae

## Abstract

Biodiversity conservation relies upon accurate species taxonomy to support decision-making. Stony corals in the genus *Pocillopora* are critical ecosystem engineers in the Eastern Tropical Pacific (ETP); however, *Pocillopora* species diversity in the region is still unresolved due to high phenotypic plasticity, lack of diagnostic morphological characters, and low-resolution genetic markers used in previous studies. To address this gap, we leveraged low-coverage whole-genome sequencing for 342 *Pocillopora* coral samples collected from Panamá, Costa Rica, Colombia, Ecuador, and Clipperton Atoll (France). Sequence data were used to recover mitochondrial genomes and barcode loci, ultraconserved elements, and genome-wide single-nucleotide polymorphisms (SNPs) for species delimitation. Together, our data revealed the existence of four distinct *Pocillopora* species in the ETP, corresponding to the nominal species *P. effusa* (Veron, 2000), *P. meandrina* Dana, 1846, *P. capitata* Verrill, 1864, and *P. lacera* Verrill, 1869. Two *P. capitata* population subclusters with moderate genetic differentiation were separated between offshore islands and continental sites, and individual colonies with signatures of admixture between *P. effusa* and *P. lacera* were identified at Isla del Coco, Costa Rica. Additionally, *Pocillopora*-associated algal symbiont community profiling identified *Cladocopium* and *Durusdinium* as dominant genera that varied according to the host species, with *P. lacera* demonstrating higher specificity for associations with *Cladocopium*. This study highlights the power of genome skimming as an affordable, high-resolution approach to rapidly assess coral species diversity and algal symbiont associations, thereby empowering marine conservation.

Significance *Pocillopora* corals are critical ecosystem engineers in the Eastern Tropical Pacific (ETP); however, accurate species identification is difficult because of high phenotypic plasticity and lack of diagnostic morphological characters. This challenge limits our understanding of species’ differences in *Pocillopora* coral ecology, adaptation, and microbial symbiosis in the ETP, and creates problems for regional efforts seeking to preserve and restore coral biodiversity. Here, we use low-coverage whole-genome sequencing to delimit ETP *Pocillopora* species. We identify four species-level genomic clusters (and one pair of population-level subclusters) that differ in their biogeographic distributions and associations with different symbiotic algae (Symbiodiniaceae) genera. This genome-based *Pocillopora* species taxonomy will be critical for future studies investigating species differences in *Pocillopora* coral ecology, physiology, adaptation, and microbial symbiosis in the ETP, and will greatly benefit efforts to monitor, conserve, and restore *Pocillopora* coral communities throughout the region.

## Introduction

Named species are often the fundamental unit of biodiversity conservation and constitute taxonomic hypotheses that rely on the criteria used to delineate their boundaries (Gaston and Mound 1993). Accurate species delimitation and taxonomic classification is essential for identifying conservation priorities that protect biologically relevant entities (Thomson et al. 2018). Speciation, or the process by which a single species diverges into two or more reproductively isolated species, is a complex and dynamic process that is influenced by organisms’ evolutionary history, biogeography, ecology, and natural selection (Mayr 1942; Palumbi 1994; Ravinet et al. 2017; Stankowski and Ravinet 2021). In marine invertebrates, classical models of allopatric speciation are challenged by observations of ongoing gene flow between populations facilitated by larval dispersal and ocean currents (Roux et al. 2016; Faria et al. 2021). Processes that can contribute to these observed patterns of “speciation with gene flow” include incomplete lineage sorting, intermittent gene flow, and population structure (see review in Ramírez-Portilla and Quattrini 2023). In addition, cryptic species, or species that are evolutionarily distinct but morphologically indistinguishable (Fišer et al. 2018), are common in marine invertebrates, as homoplasy (Forsman et al. 2009), phenotypic plasticity (Todd 2008; Paz-García et al. 2015), and morphological stasis (Flot et al. 2011; Bongaerts et al. 2021) often obscure differences between genetically distinct taxa, and the resulting lack of diagnostic morphological characters prevents accurate identification.

In reef-building corals (Order Scleractinia), these factors led to the extensive synonymizing of nominal species by coral taxonomists in the late 20th century under the assumption that morphological variation between nominal species was simply intraspecific variation within a single, widespread species (Pichon and Veron 1976). However, recent genomic approaches have uncovered substantial “cryptic” species diversity in several stony coral genera previously thought to comprise only a few geographically widespread species, such as *Acropora* (Cowman et al. 2020; Rose et al. 2021; Fifer et al. 2022; Ramírez-Portilla et al. 2022; Matias et al. 2023; Bridge et al. 2024; Rassmussen et al. 2025), *Agaricia* (Prata et al. 2022; Gijsbers et al. 2023), *Pachyseris* (Bongaerts et al. 2021; Feldman et al. 2022), *Pocillopora* (Burgess et al. 2021; Oury et al. 2023; Voolstra et al. 2023), *Porites* (Forsman et al. 2017, 2020; Starko et al. 2023; Burt et al. 2024), and *Siderastrea* (Rippe et al. 2021; Aichelman et al. 2025). Together, these results suggest that the current morphology-based taxonomy is incongruent with corals’ evolutionary relationships and underestimates the number of genetically (and potentially ecologically) distinct coral species, which inhabit smaller geographic ranges than previously thought (Bridge et al. 2024; Grupstra et al. 2024; Meziere et al. 2024; Rassmussen et al. 2025). While such studies have succeeded in documenting the boundaries between genetically distinct coral species, only a few “cryptic” coral species identified in genomic analyses have been re-examined in a formal taxonomic context.

Furthermore, sequencing methods focused on restriction-site associated DNA markers (RAD-seq) and target enrichment of ultraconserved elements (UCEs) limit our understanding of the processes that influence speciation to a reduced representation of the genome. In contrast, low-coverage whole-genome sequencing (lcWGS), also known as genome skimming (Lou et al. 2021), is an emerging method that provides high-resolution and flexible data capable of resolving species boundaries and population structure. New approaches to mine genome-skimming data for algal symbiont (Symbiodiniaceae) sequences (Ishida et al. 2024) can also be applied to uncover information about coral species’ algal symbiont associations.

*Pocillopora* Lamarck, 1816 corals are among the most geographically widespread and ecologically important corals in the world and have been the subject of foundational research on coral ecology, reproduction, cell biology, microbial symbiosis, and genomics (Glynn 1977; Stoddart 1984; Richmond 1987; Glynn et al. 1991; Hoegh-Guldberg and Williamson 1999; Ceh et al. 2011; Cunning et al. 2018). Early taxonomists in the 18th and 19th centuries described over 60 nominal *Pocillopora* species based on morphological traits, including branching patterns and polyp arrangements (World Register of Marine Species 2025). However, attempts to systematically resolve the *Pocillopora* taxonomy have been challenged by high phenotypic plasticity (Todd 2008; Paz-García et al. 2015) and morphological convergence (Pinzón et al. 2013; Marti-Puig et al. 2014). Moreover, *Pocillopora* corals demonstrate high intraspecific genetic diversity (Johnston et al. 2017) that has resulted in species being misidentified in previous studies (Johnston et al. 2018).

One development in the attempts to resolve the *Pocillopora* taxonomy was the discovery of a highly variable mitochondrial open reading frame (mtORF; Flot and Tillier 2007 ) capable of resolving several *Pocillopora* morphospecies in Hawaii and Clipperton Atoll (Flot et al. 2008, 2010). The most recent *Pocillopora* taxonomic revision (Schmidt-Roach et al. 2014) proposed links between distinct mtORF haplotypes and the nominal species *P. acuta* (Lamarck, 1816), *P. aliciae* Schmidt-Roach, Miller, & Andreakis 2013, *P. bairdi* Schmidt-Roach et al. 2014, *P.* cf. *brevicornis* (Lamarck, 1816), *P. damicornis* (Linnaeus, 1758), *P. grandis* Dana, 1846 (senior synonym of *P. eydouxi* Milne Edwards, 1860), *P. meandrina* Dana, 1846, and *P. verrucosa* (Ellis & Solander, 1786). However, this study was geographically limited to eastern Australia and the Great Barrier Reef and did not base the proposed species delimitation on genomic analyses.

Subsequent research throughout the Indo-Pacific region has continued to identify novel *Pocillopora* mtORF haplotypes (Gélin et al. 2017), and RAD-seq approaches have uncovered genome-wide divergence that indicates undescribed species diversity still exists within the genus (Johnston et al. 2022; Johnston and Burgess 2023). Most recently, a UCE bait set developed for hexacorals (Quattrini et al. 2018; Cowman et al. 2020) was applied to *Pocillopora* corals sampled throughout the Central Pacific and Western Indian Oceans, and the resulting UCE sequence data were used to delimit 15 genome-based species hypotheses (GSHs) that largely correspond to known mtORF haplotypes and nominal species (Oury et al. 2023).

In the ETP, *Pocillopora* corals are the ecologically dominant reef framework builders that provide habitat for a unique regional assemblage of fish and invertebrate species. Despite the critical importance of *Pocillopora* corals in the region, traditional taxonomy based solely on colony morphology has resulted in several species names being used by different research groups to describe different morphospecies (Glynn et al. 2017), which hampers comparisons between studies and hinders progress toward the coordination of regional conservation efforts. As a result, questions remain about the true diversity of *Pocillopora* species in the ETP, the potential for hybridization among different species lineages (Flot et al. 2010; Combosch and Vollmer 2015), and how differential associations between *Pocillopora* host species and their algal and bacterial symbionts (Cunning et al. 2013; Pettay and LaJeunesse 2013; Hernández-Zulueta et al. 2016; Palacio-Castro et al. 2023; Millán-Márquez et al. 2024) affect corals’ environmental stress tolerance. These challenges necessitate the implementation of comprehensive genomic approaches to achieve an accurate and reliable taxonomy for *Pocillopora* corals in this region.

In this study, we focused on delineating *Pocillopora* species-level genomic clusters in the ETP, and used this information to evaluate the status of the 10 *Pocillopora* morphospecies present in the region as reported in Glynn et al. (2017): *P. capitata* Verrill, 1864, *P. damicornis* (Linnaeus, 1758), *P. effusa* (Veron, 2000), *P. elegans* Dana, 1846, *P. grandis* Dana, 1846 (senior synonym of *P. eydouxi* Milne Edwards, 1860), *P. inflata* Glynn, 1999, *P. meandrina* Dana, 1846, *P. verrucosa* (Ellis & Solander, 1786), and *P. woodjonesi* Vaughan, 1918. We also evaluated the status of *P. lacera* Verrill, 1869, originally described from Panamá and previously synonymized with *P. damicornis* (Squires 1959; Reyes-Bonilla 1992).

To this aim, we used lcWGS (or genome skimming) for 342 samples collected across the ETP region to assemble mitochondrial genomes and recover mtORF barcodes, identify genome-wide SNPs, and extract UCEs for use in phylogenomic analyses. Our results reveal the existence of four distinct *Pocillopora* species-level genomic clusters (and one pair of population-level subclusters) in the ETP that match previously recorded mtORF barcodes, exhibit distinct colony morphologies, and can be assigned to GSHs and nominal species based on phylogenomic assessment of UCE loci. In addition, we leveraged noncoral reads to profile coral-associated Symbiodiniaceae communities, confirming the presence of *Cladocopium* and *Durusdinium*-dominated algal symbiont communities that varied according to the host species identity.

## Results

### Genome-skimming Data

A total of 342 samples were processed across three rounds of sequencing, with 124, 170, and 48 samples per round, respectively, producing a total of 2.92 billion sequencing reads. Samples with <1 M reads (*n* = 5) were removed, leaving 337 samples for downstream analysis (Fig. S1) with read amounts ranging from 1.1 million to 28.7 million reads (8.7 ± 4.6 million, mean ± S.D.) per sample (Fig. S2).

### Mitochondrial Genome Assembly and Analysis of Recovered mtORF Haplotypes

Mitochondrial genome assembly and annotation using MitoFinder yielded 132 complete assemblies (all predicted protein-coding genes contained on a single contig) and 205 incomplete assemblies. BLAST searches of complete and incomplete mitochondrial genome assemblies recovered the *Pocillopora* mtORF barcoding locus from 325 samples (i.e. 96.4% of samples with >1 M reads). Multiple sequence alignment revealed the existence of 5 unique mtORF haplotype sequences in the dataset, with exact matches to previously published sequences in GenBank (Table 1, Fig. S3).

**Table 1:** *Pocillopora* mtORF haplotypes recovered from mitochondrial genome assemblies and correspondence to GenBank sequences from previous studies (Pinzón et al. 2013; Gélin et al. 2017)

ORF (Gelin et al. 2017)	Corresponding lineages (Pinzon et al. 2013)	Exact GenBank sequence matches	Number of samples
27	Type 1a	HQ378758, KX538992	254
01	Type 2	HQ378759, KX538981	6
46	Type 3a	HQ378760, KX539006	61
47	Type 3b	HQ378761, KX539007	7
54	Type 3d	JX994085, KX539013	1

### Clonal Genotype Identification

Identity-by-state (IBS) analysis in ANGSD using the 3,264,865 SNPs in the full analysis dataset identified 229 unique genotypes in the dataset (using clustering height cutoffs defined by technical replicates within each mtORF type; Fig. S4). Most clonal groups were restricted to individual sites (i.e. all samples in a clonal group were found at a single site); however, one clonal group (containing the samples Coiba 679 and Coiba 931) was found across two sites (Canales, Wahoo) separated by ∼3 km on opposite sides of Isla Canales de Afuera in the Gulf of Chiriquí, and another clonal group (containing the samples Perlas 341 and Coiba 683) was found across two sites (Canales, Contadora) in the Gulf of Chiriquí and Gulf of Panamá, separated by over ∼305 km of distance.

### ETP *Pocillopora* Genomic Cluster identification

**Fig. 1.**
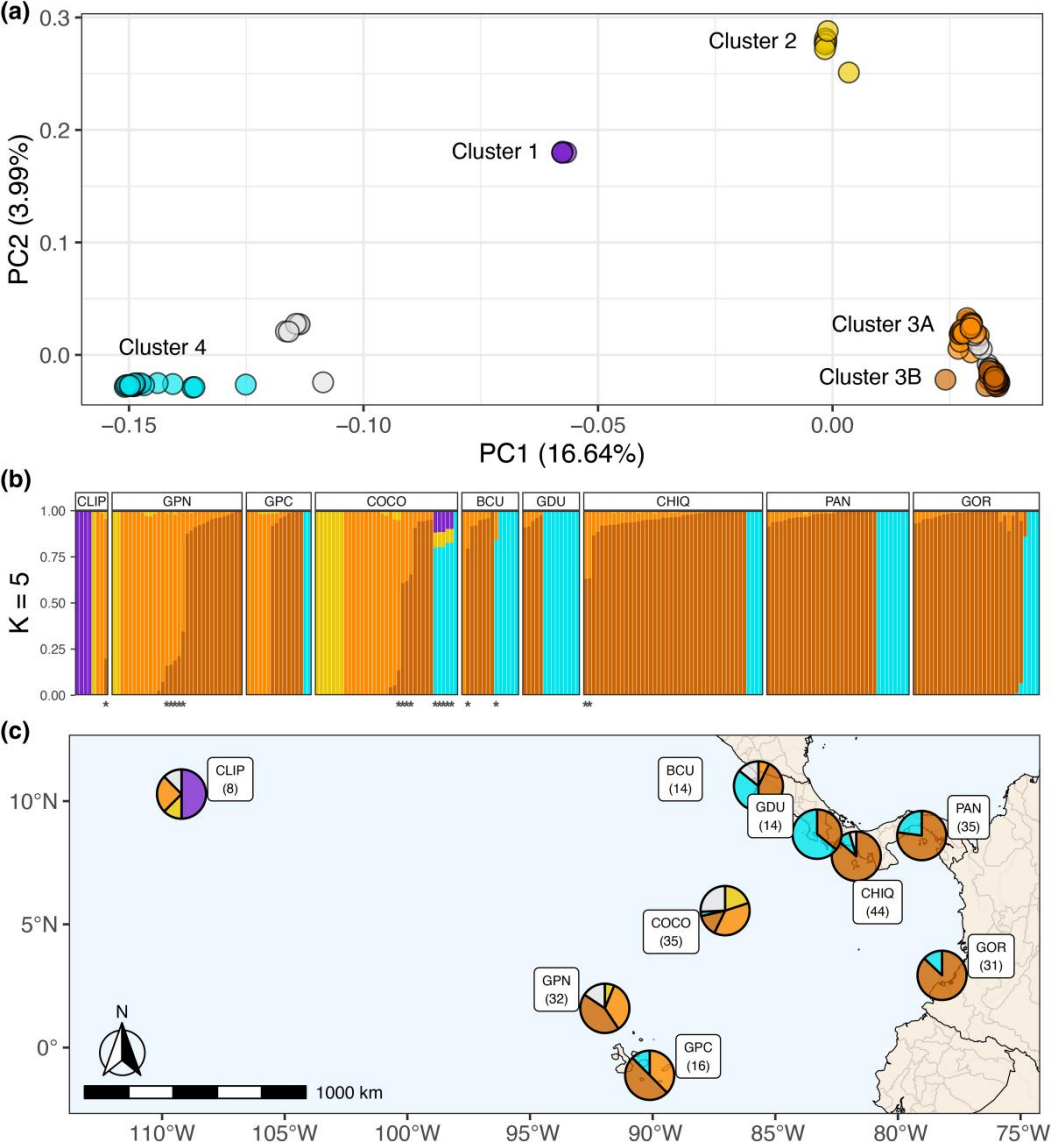
*Pocillopora* genomic cluster results summary. a) PCA of no-clones unlinked SNP dataset reveals five distinct *Pocillopora* genomic clusters. b) Bar plots of NGSadmix ancestry coefficients (q) for the most likely value of *K* = 5, unassigned samples (*q* < 0.85) are labeled with an asterisk. c) Pie charts of clusters’ relative abundances across sampling regions demonstrate differences in biogeographic distributions. Clusters are colored purple for Cluster 1, gold for Cluster 2, orange for Cluster 3A and dark orange for Cluster 3B, and turquoise for Cluster 4. Unassigned (i.e. admixed) samples are colored gray. Region codes are abbreviations for Clipperton Atoll (CLIP), Northern Galápagos (GPN), Central Galápagos (GPC), Isla del Coco (COCO), Bahía Culebra (BCU), Golfo Dulce (GDU), Gulf of Chiriquí (CHIQ), Gulf of Panamá (PAN), and Isla Gorgona (GOR), and the total number of unique genotypes for each site is listed in parentheses.

Analysis with PCAngsd and NGSadmix using the no-clones linked and unlinked SNP datasets revealed the presence of five distinct genomic clusters (Fig. 1), as K = 5 was the most likely value of K according to PCAngsd for both datasets and according to the Evanno ΔK method with NGSadmix for the unlinked SNP dataset (Fig. S5 to S9).

These genomic clusters closely matched the different observed mtORF types, with Cluster 1 containing all mtORF type 2 (ORF01) samples, Clusters 2, 3A, and 3B containing all mtORF type 1a (ORF27) samples, and Cluster 4 containing all mtORF type 3a (ORF46) samples and the lone mtORF type 3d (ORF54) sample (Table S2, Fig. S10). Samples that could not be assigned to a specific cluster (*n* = 19) were excluded from downstream phylogenetic analysis. These included 5 samples collected from Isla del Coco that were detected as admixed between Cluster 4 and Clusters 1 and 2, and all shared the unique mtORF type 3b (ORF47), and a single sample at Bahía Culebra that was admixed between Clusters 3A and 4. The other 13 samples were detected as admixed between Clusters 3A and 3B (Fig. 1), reflecting the presence of ongoing gene flow between these clusters.

Differences in the biogeographic distributions of genomic clusters were apparent, as Clusters 1 and 2 were only present at the oceanic islands of Clipperton, Isla del Coco, and Darwin and Wolf in the northern Galápagos (Fig. 1c, Table S3). Cluster 3 was separated into a pair of subclusters, with Cluster 3B dominant along the Central American continent and Cluster 3A mostly restricted to the offshore islands of Clipperton, Isla del Coco, and Galápagos (Fig. 1c). Some samples from Cluster 3A did occur in sympatry with Cluster 3B at sites at Isla del Coco and the Galápagos. Cluster 4 was more abundant along the Central American continent, with only one sample from Isla del Coco and two samples from the central Galápagos being recovered from offshore sites.

### Genomic Differentiation Among ETP *Pocillopora* clusters

Pairwise global F_ST_ indicated that these five ETP *Pocillopora* genomic clusters comprise four species-level clusters (Clusters 1–4) with strong genetic differentiation (0.266 to 0.616) and one pair of population-level subclusters (Clusters 3A and 3B) with low to moderate differentiation (0.086; Fig. S11). Pairwise F_ST_ for Cluster 3A and Cluster 4 was 0.504, indicating strong genetic isolation between these species despite their occurrence in sympatry (Fig. S11). Window-based F_ST_ indicated high genome-wide divergence between all species-level genomic clusters, compared with lower F_ST_ between population-level subclusters 3A and 3B (Fig. S12).

### Phylogenetic Analysis of UCE Loci Confirms Systematic Relationships

**Fig. 2.**
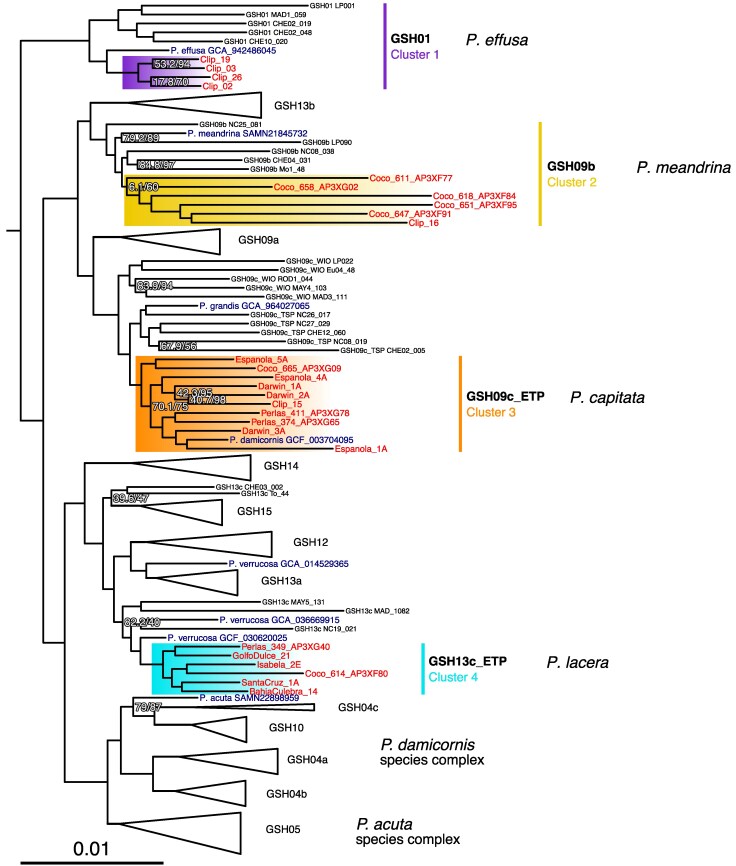
Systematic relationships of ETP *Pocillopora* species-level genomic clusters (highlighted in color) revealed by UCE phylogenomic analysis. ML phylogenetic tree of UCE loci recovered from representative samples of ETP species-level genomic clusters (highlighted in color), previously identified GSHs from Oury et al. (2023), and *Pocillopora* reference genomes (labeled in dark blue). The tree was constructed using a 50% data matrix with 138 samples, 1,513 UCE loci, and 717,549 bp. Nodes with <90% ultrafast bootstrap (UFBoot) support are labeled with UFBoot, and SH-aLRT test support values are shown as UFBoot/SH-aLRT.

Bioinformatic extraction of UCE loci from genome-skim data enabled comparisons of these ETP *Pocillopora* species-level genomic clusters with previously published *Pocillopora* GSHs (Oury et al. 2023) and eight reference genomes (Fig. S13). After processing using the PHYLUCE pipeline, the 50% data matrix (comprising UCEs in at least 50% of samples) contained 1,513 UCE loci with 717,549 bp and 194,624 informative sites, and the 75% data matrix contained 1,263 UCE loci with 613,088 bp and 166,712 informative sites. Maximum likelihood phylogenetic analysis using IQ-TREE2 produced for both datasets highly similar tree topologies that recovered all previously described *Pocillopora* GSHs as well-resolved monophyletic clades with high node support (Fig. 2, Fig. S14 to S16). This permitted the assignment of the four *Pocillopora* ETP species-level genomic clusters to previously described GSHs and associated nominal species (Fig. 2, Table 2).

**Table 2:** Correspondence between ETP *Pocillopora* species-level genomic clusters and previously described mtORF types and GSHs

Cluster	Corresponding lineages (Flot et al. 2010)	Corresponding lineages (Schmidt-Roach et al. 2013)	Corresponding lineages (Pinzón et al. 2013)	ORF haplotypes (Gelin et al. 2017)	GSH (Oury et al. 2023)	Species identification (Oury et al. 2023)	ETP *Pocillopora* species (this study)
1	*Pocillopora* sp. B	N/A	Type 2	01	GSH01	*P. effusa*	*P. effusa* (Veron, 2000)
2	*Pocillopora* sp. A	*P. meandrina*	Type 1a	27	GSH09b	*P. meandrina*	*P. meandrina* Dana, 1846
3A/3B					GSH09c_ETP	*P. grandis*	*P. capitata* Verrill, 1864
4	N/A	Type γ (“gamma”)	Type 3a, Type 3d	46, 54	GSH13c_ETP	*P. verrucosa*	*P. lacera* Verrill, 1869

Cluster 1 samples formed a clade within GSH01, corresponding to *P. effusa* (Veron, 2000), while Cluster 2 samples were grouped in GSH09b, or *P. meandrina* Dana, 1846. Samples from Cluster 3A and 3B formed a clade within GSH09c, currently termed *P. grandis* Dana, 1846, that was sister to a clade containing samples from the tropical South Pacific (GSH09c_TSP; Fig. 2). This new clade, which we term GSH09c_ETP and identify as the nominal species *P. capitata* Verrill, 1864, contains samples from both Clusters 3A and 3B, but these were not recovered as reciprocally monophyletic. Additionally, all the Cluster 4 samples formed a separate, well-supported clade within GSH13c, currently termed *P. verrucosa* (Ellis & Solander, 1786), which we term GSH13c_ETP and identify as the nominal species *P. lacera* Verrill, 1869 (see Taxonomic Account, Taxonomic Account Figs. 1 and 2).

Notably, of the eight *Pocillopora* reference genomes included in the phylogeny, three appear to be misidentified according to the current taxonomy. *P. “damicornis*” GCF_003704095 (Cunning et al. 2018) was grouped within the ETP *P. capitata* clade (GSH09c_ETP), and *P. “acuta*” SAMN22898959 was grouped in a clade with samples of GSH04c and GSH10, which currently lack formal taxonomic descriptions but are not considered *P. acuta* sensu stricto (Oury et al. 2023). Our results also group *P. “verrucosa*” GCA_014529365 from the Red Sea with GSH13a, recently described as *P. favosa* (Oury et al. 2025).

### Comparison of Species-level Genomic Clusters With Nominal Species Descriptions

**Fig. 3.**
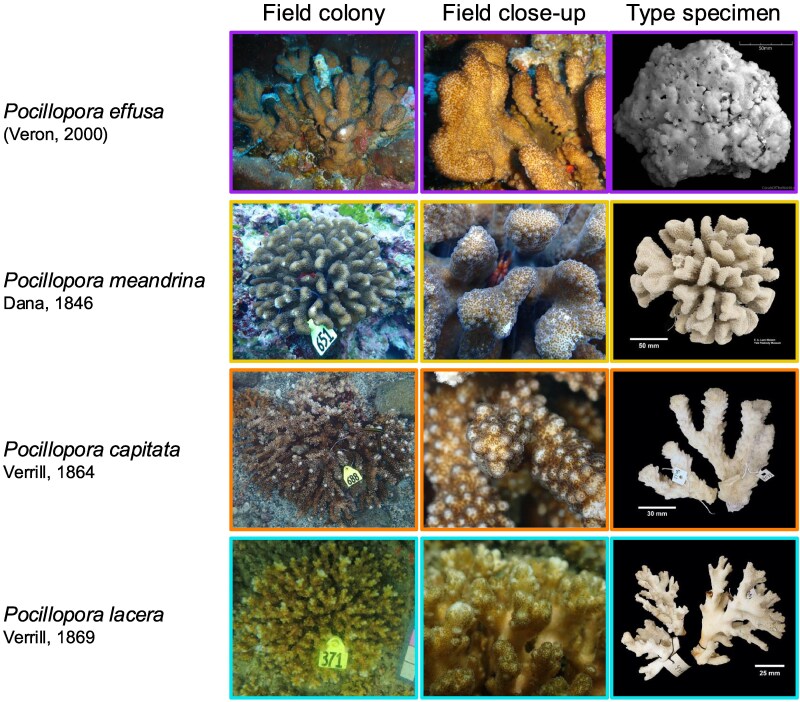
Representative field photographs of colony morphologies for the ETP *Pocillopora* putative species-level genomic clusters and images of type specimens for the proposed species name of each cluster. Type specimen images for each nominal species are from the following specimens: *P. effusa* (MTQ G55781), *P. meandrina* (YPM IZ 001970.CN), *P. capitata* (YPM IZ 004033.CN), and *P. lacera* (YPM IZ 004490.CNB).

Based on morphological examination of field photographs and skeletal specimens from our study in comparison to type material and original descriptions, the four species-level genomic clusters were identified as the nominal species *P. effusa* (Veron, 2000) for Cluster 1, *P. meandrina* Dana, 1846 for Cluster 2, and *P. capitata* Verrill, 1864 for Cluster 3. Cluster 4 most closely corresponded to *P. lacera* Verrill, 1869, which is currently considered a junior synonym of *P. damicornis* (Linnaeus, 1758; Squires 1959; Reyes-Bonilla 1992; Fig. 3; see Taxonomic Account).

### Symbiodiniaceae Associations Vary According to Host Species and Geography

**Fig. 4.**
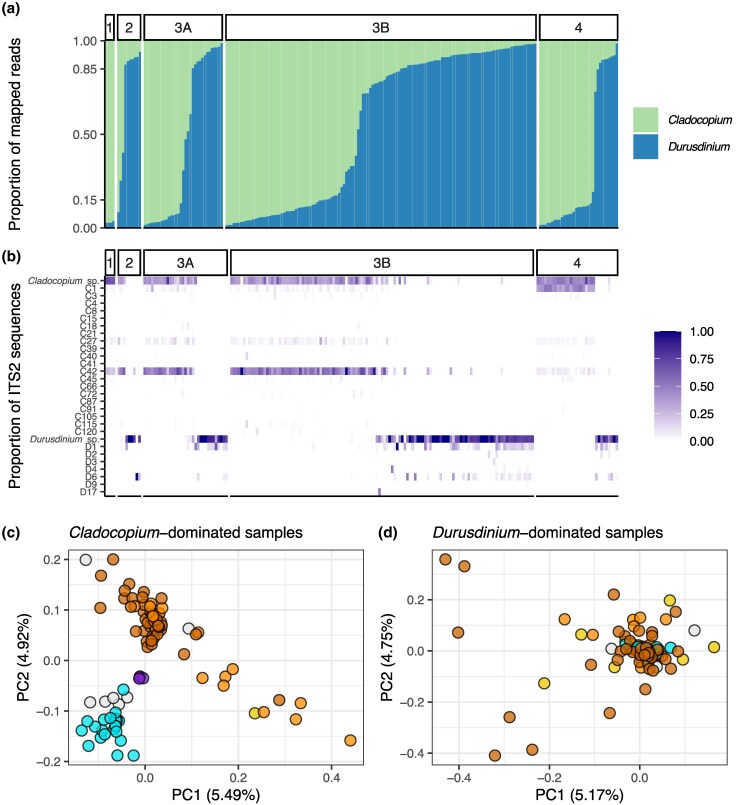
*Pocillopora* symbiodiniaceae community profiling reveals *Cladocopium*, *Durusdinium*, and mixed-dominance associations, with greater host specificity in *Cladocopium*-dominated samples. a) Barplot of the proportion of noncoral reads mapping to the *Cladocopium* (green) versus *Durusdinium* (blue) references, ordered according to the highest proportion of *Cladocopium* and facetted by *Pocillopora* species-level clusters. b) Heatmap of the proportion of ITS2 sequences recovered from noncoral reads using graftM. The rows *Cladocopium* sp. and *Durusdinium* sp. represent ITS2 sequences that did not match a specific reference sequence but could still be assigned to either genus. PCA plots of c) *Cladocopium*-dominated samples based on 1,279 *Cladocopium* SNPs and d) *Durusdinium*-dominated samples based on 1,699 *Durusdinium* SNPs recovered with ANGSD, respectively. The *Pocillopora*-host genomic clusters are colored as in Fig. 1.

Alignment of 184 million noncoral reads from the 229 “no-clones” samples against the concatenated *Cladocopium* and *Durusdinium* reference genomes identified 92 samples as *Cladocopium*-dominated, 92 samples as *Durusdinium*-dominated, and 45 samples as having mixed-dominance Symbiodiniaceae communities (Fig. 4a). ITS2 sequences recovered by graftM confirmed these associations, with “*Cladocopium* sp”, “C1”, “C27” and “C42” being the most common *Cladocopium*-associated sequences, and “*Durusdinium* sp”, “D1” and “D6” as the most common *Durusdinium*-associated sequences (Fig. 4b).

*Pocillopora* species-level genomic clusters differed in their Symbiodiniaceae community associations. Cluster 1 (*P. effusa*) was entirely *Cladocopium*-dominated (4/4 samples), while Cluster 2 (*P. meandrina*) was mostly *Durusdinium*-dominated (7/10 samples), with one *Cladocopium*-dominated specimen and two mixed communities (Fig. 4a). Clusters 3A and 3B (*P. capitata*) demonstrated greater variability in Symbiodiniaceae associations, with many *Cladocopium*-dominated (57/163 samples), *Durusdinium*-dominated (70/163 samples), and mixed algal symbiont communities (36/163 samples). Cluster 4 (*P. lacera*) also possessed variable associations, albeit with a greater proportion of *Cladocopium*-dominated (22/33 samples) than *Durusdinium*-dominated (8/33 samples), and much fewer mixed communities (3/33 samples).

Interestingly, PCA analysis of SNPs recovered from *Cladocopium* and *Durusdinium*-dominated samples revealed Symbiodiniaceae community clusters that more closely corresponded to coral-host genomic clusters for *Cladocopium* (Fig. 4c) than for *Durusdinium* (Fig. 4d), suggesting that *Pocillopora* coral hosts maintain more specific associations with *Cladocopium* symbionts than *Durusdinium* symbionts. The recovered ITS2 sequences also suggest specific *Pocillopora*-*Cladocopium* associations, as “C27” and “C42” were most common in Clusters 1, 2, 3A and 3B, and “C1” was most common in Cluster 4 (Fig. 4b). These ITS2 types are representative of the different *Cladocopium* species *C. latusorum* and *C. pacificum*, respectively, which are known to differ in their associations with *Pocillopora* coral hosts (Turnham et al. 2021). In contrast, the most common *Durusdinium*-associated ITS2 sequences “D1” and “D6” indicate that *D. glynnii* (Wham et al. 2017) is the only *Durusdinium* species present in our dataset.

Further examination of biogeographic patterns in algal symbiont associations within *P. capitata* and *P. lacera* revealed a trend toward greater prevalence of *Durusdinium*-dominated samples in sites along the Central American continent as opposed to offshore islands (Fig. S17). In general, Golfo Dulce in Costa Rica had the highest prevalence of *Durusdinium*-dominated samples in both species (10/14 samples), while other sites with a high prevalence of *Durusdinium*-dominated samples, including Bahía Culebra in Costa Rica (9/14 samples) and Isla Gorgona in Colombia (15/31 samples; Fig. S17).

## Discussion

In this study, we used genome-skimming and phylogenomic analyses to delimit genomic clusters among *Pocillopora* specimens collected in the ETP, revealing the presence of four species-level clusters (including one pair of population-level subclusters) that we tentatively attributed to the nominal species *P. effusa*, *P. meandrina*, *P. capitata*, and *P. lacera*. We document clonal populations of *P. capitata* and *P. lacera*, the two dominant *Pocillopora* species along the Central American continent. Notably, there are clear biogeographic differences in the distribution patterns of these species across the region, highlighting the unique environmental and ecological factors influencing their habitats. We observed substantial population differentiation between *P. capitata* populations at continental sites and those located on offshore islands, suggesting isolation and local adaptation, punctuated by intermittent gene flow, among continental and offshore corals. This differentiation underscores the importance of oceanographic barriers and connectivity corridors in shaping coral genetic structure in the ETP (Baums et al. 2012). Additionally, our study demonstrates the efficacy of using lcWGS for coral holobiont genomic characterization (including Symbiodiniaceae community profiling), providing a robust and cost-effective approach for future research in marine genomics. These insights are crucial for understanding the evolutionary processes and conservation strategies necessary for these ecologically vital coral species.

### Distinct *Pocillopora* Genomic Species are Present in the ETP region

Our genomic analysis of *Pocillopora* species in the ETP confirms the presence of four distinct species-level genomic clusters across the region. These lineages were tentatively identified as *P. effusa* (Veron, 2000), *P. meandrina* Dana, 1846, *P. capitata* Verrill, 1864, and *P. lacera* Verrill, 1869, with *P. capitata* and *P. lacera* established as the dominant species present along the Central and South American continent. These two species exhibit high levels of clonality and low intraspecific genetic differentiation, indicative of strong population connectivity among continental populations. In contrast, total *Pocillopora* species diversity increases at offshore islands, with Isla del Coco, the Galápagos, and Clipperton recognized as biodiversity hotspots within the region.

Notably, our analysis did not recover evidence for the presence of *P. damicornis* (Linnaeus, 1758), *P. elegans* Dana, 1846, *P. inflata* Glynn, 1999, and *P. woodjonesi* Vaughan, 1918 using either genomic or morphological examination. The apparent absence of these species, despite their historical records in the region (Combosch et al. 2008; Glynn et al. 2017; Lizcano-Sandoval et al. 2018), could be due to local extinctions, shifts in species distributions, or a lack of suitable sampling locations where these species might still exist. However, we consider it likely that the high morphological plasticity in *Pocillopora* species and the incomplete taxonomic resolution in previous studies led to species misidentifications, and several of these species (i.e. *P. elegans*, *P. inflata*, and *P. woodjonesi*) do not exist as discrete genetic entities in the ETP as previously thought. Of these nominal species, only *P. inflata* has its type locality in the ETP; the type localities of *P. elegans* and *P. woodjonesi* are in Fiji and the Cocos (Keeling) Islands, respectively. As recent genomic and morphological analysis in *Acropora* has revealed that coral species can have more discrete ranges than previously assumed (Cowman et al. 2020; Bridge et al. 2024; Rassmussen et al. 2025), further research and broader sampling efforts that specifically target type localities are necessary to clarify the status and distribution of these species (Bonito et al. 2021).

Cluster 4 presents a unique case in our analysis, as it belongs to the *Pocillopora verrucosa* clade according to Oury et al. (2023), yet exhibits distinct characteristics that set it apart from other members of this species present in different geographic regions. Specifically, individuals in Cluster 4 (GSH13c_ETP) possess the unique mtORF type 3a (ORF46), a genetic marker attributed to the *P. verrucosa* clade (Schmidt-Roach et al. 2014). However, the neotype of *P. verrucosa* collected from Lizard Island on the Great Barrier Reef does not possess this specific haplotype. ORF46 is also diagnostic of *P. villosa*, or GSH13b (Oury et al. 2022, 2023), where it occurs in the western Indian Ocean; however, the UCE phylogenomic data place *P. villosa* in a separate clade sister to *P. meandrina*, while Cluster 4 samples form a clade (GSH13c_ETP) within the *P. verrucosa* species complex (Fig. 2). This case of apparent convergent evolution could instead be due to ancestral mitochondrial introgression, aka mitochondrial genome capture (Mastrantonio et al. 2019). This pattern of mitonuclear discordance underscores the value of genome-wide sequencing for resolving species boundaries and phylogenetic relationships (Quattrini et al. 2023). Additionally, field images and voucher specimens for Cluster 4 samples show strong morphological similarities to *P. lacera* Verrill, 1869, which was originally described from Panamá. Later studies erroneously identified this morphology as *P. damicornis* (Linnaeus, 1758) in the ETP (Squires 1959; Reyes-Bonilla 1992), leading to its incorrect synonymization (Pichon and Veron 1976). The presence of a diagnostic mitochondrial haplotype alongside its unusual morphology and its monophyly observed in the UCE phylogenetic tree suggests that these individuals represent a distinct species lineage within the *P. verrucosa* complex (see Taxonomic Account).

Finally, the detection of colonies with signatures of introgressive hybridization at Isla del Coco is particularly noteworthy, as it suggests that hybridization may play a role in maintaining or enhancing genetic diversity within isolated coral populations (Forsman et al. 2017; Meziere et al. 2024). At present, these five individuals appear to be hybrids between *P. lacera* (Cluster 4) and another species such as *P. effusa* or *P. meandrina* (Clusters 1 and 2), but further study will be required to characterize these colonies’ ancestry. Conversely, only one sample from Bahia Culebra was detected as admixed between offshore *P. capitata* and *P. lacera* (the only case of admixture between mtORF types detected in our study), indicating that inter-species hybridization is not as common between *Pocillopora* species as was previously suggested (Combosch et al. 2008).

### Considerations for Taxonomic Identification of Coral Reference Genomes

Our analysis of *Pocillopora* UCE loci revealed that at least three of the eight *Pocillopora* reference genomes included in the phylogeny were misidentified according to the current taxonomy. Our results identify *P.* “*damicornis*” GCF_003704095 (Cunning et al. 2018) as *P. capitata* (GSH09c_ETP) and group *P.* “*acuta*” SAMN22898959 with GSH04c and GSH10, which currently lack taxonomic descriptions (Oury et al. 2023). Furthermore, our results group the *P.* “*verrucosa*” GCA_014529365 from the Red Sea with GSH13a, recently described as *P. favosa* (Oury et al. 2025). To avoid similar confusion in the future, we encourage research groups to contextualize new reference genome assemblies according to the most recent taxonomy and known phylogenomic relationships. For example, Bridge et al. (2024) included genomes in their *Acropora* phylogenomic study, demonstrating several misidentified genomes in that speciose genus. Therefore, we suggest incorporating integrative taxonomy into genome studies and following recommendations by Voolstra et al. (2021).

### *Pocillopora* Associations With *Cladocopium* and *Durusdinium* Algal Symbionts Vary According to Host Species and geography

Previous studies have shown that host species identity and environmental context jointly shape *Pocillopora* corals’ Symbiodiniaceae associations in the ETP (Pinzón and Lajeunesse 2011; Pettay and LaJeunesse 2013; Turnham et al. 2021; Palacio-Castro et al. 2023; Millán-Márquez et al. 2024). Recently, Palacio-Castro et al. (2023) and Millán-Márquez et al. (2024) found that different *Pocillopora* lineages (identified using the mtORF barcode) specifically associate with different *Cladocopium* species, *C. latusorum* and *C. pacificum*, across the ETP. However, these associations were flexible and shifted to *Durusdinium*-dominated assemblages in environments marked by high sea surface temperatures and turbidity.

These findings are consistent with the results of our analysis of genome skimming data and suggest that specific *Pocillopora*-*Cladocopium* combinations are ecologically and evolutionarily stable in the ETP, with *P. capitata*, *P. effusa* and *P. meandrina* broadly associating with *C. latusorum*, and *P. lacera* specifically associating with *C. pacificum*. However, these associations, especially with *C. latusorum*, can shift toward *Durusdinium* dominance in environments with high sea surface temperatures or turbidity (Baker et al. 2013; Palacio-Castro et al. 2023; Millán-Márquez et al. 2024; Glynn et al. 2025). For example, the greatest prevalence of *Durusdinium*-dominated *P. lacera* in our study was found in Golfo Dulce, Costa Rica, an enclosed tropical fjord with high levels of terrestrial sediment input and a recent history of severe bleaching (Cortés 1990; Alvarado et al. 2020). Such specificity may reflect long-term co-evolutionary relationships or functional compatibility that enhances holobiont performance under local environmental regimes, while also maintaining enough flexibility to switch to a more beneficial algal symbiont during sudden environmental stress (Cunning et al. 2015; Abbott et al. 2021; Ros et al. 2021; Turnham et al. 2023). From the algal symbiont perspective, these patterns of host specificity may arise because vertical transmission of *Cladocopium* in oocytes, which has been observed in ETP *Pocillopora* (Glynn et al. 1991), increases the efficiency of natural selection at favoring beneficial symbionts and enhancing rates of co-diversification (Bennett and Moran 2015). In contrast, as a generalist species, *D. glynnii* maintains associations with corals even during periods of environmental stress (Baker et al. 2013) and, being horizontally acquired from an environmental pool of free-living algae, occupies the vacant niches that become available following bleaching events (Glynn et al. 2001).

### Implications for Coral Conservation and Genomics-guided Management Strategies in the ETP

The discovery of distinct *Pocillopora* species and the presence of high levels of clonality and population connectivity in continental populations contrast significantly with the more genetically diverse and isolated populations at offshore island locations such as Clipperton Atoll, Isla del Coco, and the Galápagos (Fig. 1). These findings suggest that different conservation strategies are required for island versus continental populations. Offshore island populations, with their unique genetic diversity and smaller population sizes, may be more vulnerable to localized extinction events due to their isolation and reduced connectivity to other populations (Underwood et al. 2020). Conservation efforts in these areas should prioritize the protection of genetic diversity and the preservation of habitat integrity to prevent genetic bottlenecks.

In contrast, continental populations with higher clonality may benefit more from strategies aimed at maintaining population connectivity and density and mitigating local disturbances, such as coastal development or pollution (Feldman et al. 2022). Fine-scale geographic sampling of continental *P. capitata* and *P. lacera* populations may aid in identifying breaks in genetic connectivity, as previous work suggests that limits to gene flow exist at scales <35 km (Underwood et al. 2020). Identification of these clines in genetic diversity along the Central American continent through fine-scale sampling may highlight opportunities for assisted gene flow via migration of nursery-reared colonies (Baums et al. 2019).

Genomics-guided management, which can identify genetically important populations and track introgression and connectivity, will be crucial in designing tailored conservation strategies (Pinsky et al. 2023). Furthermore, the regional differences in genetic diversity highlight the need for international collaboration to ensure the effective protection of coral populations across the ETP, particularly in areas such as Isla del Coco, the Galápagos, and Clipperton that are *Pocillopora* biodiversity hotspots.

## Conclusion

Our genomic analysis of ETP *Pocillopora* corals reveals significant coral host genetic and algal symbiont community diversity, with distinct geographic distributions of the nominal species *P. effusa*, *P. meandrina*, *P. capitata,* and *P. lacera* among continental and offshore island sites. The high clonality observed in continental populations contrasts with the increased genetic diversity and signatures of historical introgression in isolated island populations such as Clipperton Atoll, Isla del Coco, and the Galápagos Islands. These differences underscore the need for region-specific conservation strategies, with island populations requiring focused efforts on genetic preservation, while continental populations may benefit from connectivity and habitat protection measures. Genomics-guided management will be essential to maintaining coral biodiversity and resilience in the face of increasing environmental pressures across the ETP.

## Materials and Methods

### Sample Collection and DNA Extraction

A primary set of *Pocillopora* coral tissue samples and skeleton voucher specimens was collected from Smithsonian Tropical Research Institute (STRI) monitoring sites located in the Gulf of Panamá and Gulf of Chiriquí in the tropical eastern Pacific of Panamá. Field photographs were obtained for each sampled colony and 1 cm^2^ tissue fragments were preserved in Zymo DNA/RNA Shield using stainless steel bone cutters that were rinsed with 70% ethanol and DI water between each sample. Corresponding skeleton voucher specimens were collected, air dried, and accessioned into the Invertebrate Zoology collection at the Smithsonian National Museum of Natural History (NMNH, USNM1666022–USNM1666110). DNA was extracted from the coral tissue samples using the standard phenol-chloroform protocol on the Autogen GENE PREP instrument (https://autogen.com/product/gene-prep/), with an additional ethanol wash step to eliminate phenol carryover.

Additional coral samples were collected from sites in Costa Rica (Golfo Dulce, Bahía Culebra, and Isla del Coco), Colombia (Isla Gorgona), Ecuador (Galápagos Islands: Santa Cruz, San Cristóbal, Española, Floreana, Isabela, Wolf, and Darwin) and Clipperton Atoll (France; Flot et al. 2010). Genomic DNA was extracted from these samples using the Qiagen DNeasy Blood & Tissue kit or phenol-chloroform methods and shipped to the Smithsonian NMNH for inclusion in molecular lab work (Table S1).

### Library Preparation and Sequencing

DNA concentration for all samples was quantified using the Qubit dsDNA HS kit and DNA fragment size distributions were checked using electrophoresis on a 1.5% agarose gel. Genomic libraries were prepared using the NEBNext Ultra FS II kit with ½ reaction volumes and amplified for 10X PCR cycles with the KAPA HiFi HotStart mix. Library concentrations were quantified using the Qubit dsDNA HS kit, and size distributions were checked using the Agilent TapeStation 4000 instrument and HS D1000 kit. Successful libraries were normalized to a target concentration of 4 nM and pooled before being shipped to an external facility for sequencing to a target depth of 10 million 150-bp paired-end reads per sample.

### Read Processing, Mitochondrial Genome Assembly and mtORF Barcode Recovery

Raw reads were checked for sufficient read depth (>1 M reads) and quality scores using FASTQC and low-quality reads were trimmed and adapter sequences removed using Trimmomatic v0.39 (Bolger et al. 2014; Fig. S1). Trimmed reads were used to assemble mitochondrial genomes for each sample using MitoFinder v1.4.1 (Allio et al. 2020), using *Pocillopora* mitochondrial genomes available in GenBank (NC009797, NC009798) as references (Flot and Tillier 2007). BLAST v2.10.1 was used to search the resulting complete and incomplete mitochondrial genome assemblies using the *Pocillopora* mtORF locus (HQ378760) as a query. Best-hit subject sequences were extracted and aligned with MAFFT (Katoh and Standley 2013) and visualized as a median-joining haplotype network using the R package “pegas” (Paradis 2010). All recovered mtORF haplotypes were matched to previously published sequences in GenBank (Pinzón et al. 2013; Gélin et al. 2017) and used to assign correspondence with previous nomenclature (Table 1). To generate high-quality mitochondrial reference genomes, complete assemblies from representative samples of each mtORF type were circularized using SimpleCircularise (https://github.com/Kzra/Simple-Circularise) and circular contigs were re-annotated using MitoFinder in assembly mode.

### Reference Genome Alignment and Clonal Genotype Identification

Trimmed reads were aligned to the chromosome-scale *Pocillopora grandis* reference genome (GCA_964027065.2) using BWA v0.7.17 (Li and Durbin 2009). Aligned reads were converted to BAM files, sorted and indexed using samtools v1.17 (Li et al. 2009) and Picard v2.20.6 was used to add read groups and mark PCR duplicate reads with AddOrReplaceReadGroups and MarkDuplicates. MultiQC v1.9 (Ewels et al. 2016) was used to evaluate pipeline performance (Fig. S2).

To identify clones, we used the single-read sampling approach in ANGSD v0.940 (Korneliussen et al. 2014) to compute IBS as in Manzello et al. (2019). The input BAM files were filtered for a minimum mapping quality of 30, minimum base quality score of 30, at least 85% nonmissing genotypes, a minimum minor allele frequency (MAF) of 0.05, and a SNP *P*-value of 10^−5^. Additional filters were used to remove reads with multiple best hits, SAM flags above 255, sites with high strand bias and tri-allelic sites, leaving 3,264,865 SNPs. Pairwise IBS distances were clustered using the function hclust() in R and the resulting dendrogram was visualized using the R package “ggtree” (Yu et al. 2017). Technical replicates from each mtORF type were sequenced to establish the appropriate clustering height cutoffs for within-individual genetic similarity and identify samples belonging to clonal genotypes (Manzello et al. 2019; Fifer et al. 2022; Matias et al. 2023). Only one sample with the highest proportion of reads with at least 5X sequencing depth from each clone group was retained to create a “no-clones” set of 229 samples (i.e. unique genotypes) for use in downstream analyses (Shilling et al. 2023; Eckert et al. 2024).

### Species Delimitation and Differentiation Among *Pocillopora* Genomic clusters

ANGSD was used to apply the same filters described above to BAM files in the no-clones sample set, resulting in 5,587,480 linked SNPs. These linked SNPs were pruned for linkage disequilibrium using ngsLD (Fox et al. 2019) and prune_graph (https://github.com/fgvieira/prune_graph) with max distance <10 kb and minimum r^2^ weight >0.5 for each genomic scaffold, leaving 1,310,435 unlinked SNPs (Fig. S1). PCAngsd (Meisner and Albrechtsen 2018) and NGSadmix (Skotte et al. 2013), which operate on genotype likelihoods rather than hard-called SNPs, were used to identify genomic clusters in the no-clones linked and unlinked SNP datasets. For both datasets, PCAngsd identified the most likely value of K = 5. The NGSadmix analyses were conducted for values of K ranging from 1 to 10, and the most likely value of K = 5 was determined for unlinked SNP dataset using the Evanno ΔK method implemented in R (Evanno et al. 2005).

To assess genomic differentiation among clusters, we identified samples that were representative of their clusters (NGSadmix K = 5 assignment probability q ≥ 0.85) and selected the top five samples with the highest proportion of >5X sequencing depth for each cluster (*n* = 24) as “representative” samples. Genotype likelihoods were calculated in ANGSD for the representative samples using the same filters described above to retain sites in the unlinked SNP dataset. To determine genetic differentiation between genomic clusters, ANGSD was used to calculate the site allele frequency (SAF) for each cluster, and then realSFS calculated the site frequency spectrum (SFS) for all possible pairwise comparisons between clusters. These SFSs were used as priors with the SAF to calculate global F_ST_ and 50 kb window-based F_ST_. Here, weighted global F_ST_ estimates between pairwise comparisons of the genomic clusters are reported.

### UCE Phylogenomics Using Genome-skim Data

To contextualize the taxonomic relationships of the genomic clusters, we included select samples from specific locations for certain clusters (i.e. Clip_16 for Cluster 2, Coco_614 for Cluster 4) with the representative samples for UCE phylogenomic analysis. Briefly, trimmed reads were assembled into contigs using SPAdes v3.14.0 (Bankevich et al. 2012) and UCE loci were extracted *in silico* using the PHYLUCE pipeline (Faircloth 2016) and the *hexa-v2-final* probe set (Cowman et al. 2020). Target enrichment sequencing reads for five samples from each of the *Pocillopora* GSHs identified in Oury et al. (2023) were downloaded from the NCBI SRA and processed alongside the TEP representative samples, and UCEs were bioinformatically harvested from 8 publicly available *Pocillopora* reference genomes (Cunning et al. 2018; Buitrago-López et al. 2020; Stephens et al. 2022; Noel et al. 2023) to further contextualize taxonomic relationships. UCE loci from *Stylophora pistillata* GSH samples and the *S. pistillata* reference genome (Voolstra et al. 2017) were included as outgroups. Extracted UCE loci were internally trimmed using GBLOCKS v0.91b (Castresana 2000), aligned using MAFFT, and concatenated into a PHYLIP-formatted alignment. Maximum likelihood phylogenetic inference was performed using IQ-TREE2 (Minh et al. 2020) on a partitioned dataset, with ModelFinder (Kalyaanamoorthy et al. 2017) for the selection of the best-fit partition scheme and nucleotide substitution model. The phylogenetic trees were inferred with UltraFast (UF) bootstrap replicates to assess branch support (Minh et al. 2013; Hoang et al. 2018) and the resulting phylogenetic trees were visualized using the R package “ggtree”.

### Profiling Symbiodiniaceae Community Associations Using Genome-skim Data

*Pocillopora* corals in the ETP have been shown to associate with algal symbionts (Family Symbiodiniaceae) belonging to the genera *Cladocopium* and *Durusdinium* (Cunning et al. 2013; Palacio-Castro et al. 2023; Millán-Márquez et al. 2024). To determine the dominant Symbiodiniaceae genera in each sample, paired reads that did not map to the *Pocillopora* reference genome were extracted using the samtools bam2fastq command, and the resulting noncoral reads (mean 932,805 reads, approximately 2% to 9% from each sample) were aligned to a concatenated reference containing the genome sequences of *Cladocopium goreaui* (Liu et al. 2018) and *Durusdinium trenchii* (Shoguchi et al. 2021). Reference genomes for *Symbiodinium*, *Breviolum*, or other Symbiodiniaceae genera were not included in the concatenated reference because these genera have not been previously reported to frequently associate with *Pocillopora* corals in the ETP (Palacio-Castro et al. 2023; Millán-Márquez et al. 2024; Glynn et al. 2025). A custom perl script zooxtype.pl (Manzello et al. 2019) was used to count the number of reads mapped to either reference genome, and was used as a proxy to determine the relative abundance of *Cladocopium* versus *Durusdinium* symbionts in each sample. Samples with a greater than 85% proportion of reads mapping to a single genus were considered as *Cladocopium*-dominated or *Durusdinium*-dominated, respectively. To validate these genus-level results, ITS2 sequences of Symbiodiniaceae were extracted from noncoral reads with graftM (Boyd et al. 2018) using a custom ITS2 library assembled from the SymPortal database (Hume et al. 2019; Ishida et al. 2024).

To examine within-genus diversity, genome-wide SNPs were identified using ANGSD by analyzing *Cladocopium*-dominated samples and *Durusdinium*-dominated samples separately, retaining only reads that mapped to their respective reference genomes with a minimum mapping quality of 20 and minimum base quality score of 20, with the rest of the filters the same as the coral host analysis (Eriksson et al. 2025). PCAngsd was used to identify clusters of within-genus algal symbiont diversity that associated with host species-level clusters based on 1,279 SNPs for *Cladocopium* and 1,699 SNPs for *Durusdinium*, respectively.

## Supplementary Material

evaf235_Supplementary_Data

## Data Availability

Raw Illumina whole-genome sequencing reads were deposited into the NCBI Sequence Read Archive (SRA) under BioProject accession PRJNA1055517. All the scripts used in bioinformatic data analysis are available on GitHub: https://github.com/michaeltconnelly/etp_pocillopora_gskim.
