# Supplementary material for: Genome Skimming Illuminates Hidden Species Diversity and Symbiodiniaceae Associations in East Pacific Pocillopora Corals
Source: Genome Biol Evol. 2025 Dec 3;18(2):evaf235. doi: 10.1093/gbe/evaf235 (PMC12859748; doi:10.1093/gbe/evaf235)
Supplement: evaf235_Supplementary_Data [file evaf235_supplementary_data.zip › SupplementaryMaterials_PociGSkim_111725.pdf]

Supplementary Material for “**Genome skimming illuminates hidden species diversity and Symbiodiniaceae associations in east Pacific *Pocillopora* corals**”

Table S1: *Pocillopora* coral sampling regions, sites, coordinates, and sample numbers.

Country	Region	Code	Latitude	Longitude	*N*
France	Clipperton Atoll	CLIP	10.28714	-109.20635	10
Costa Rica	Bahía Culebra	BCU	10.61151	-85.68752	29
	Golfo Dulce	GDU	8.64895	-83.28039	19
	Isla del Coco	COCO	5.55533	-87.04648	39
Panamá	Bahía Panamá, Islas Perlas	PAN	8.61240	-79.04165	75
	Bahía Chiriquí, Isla Coiba	CHIQ	7.77281	-81.71475	51
Colombia	Isla Gorgona	GOR	2.92975	-78.20268	53
Ecuador	Galápagos Northern	GPN	1.58978	-91.94836	35
	Galápagos Central/Southern	GPS	-1.04288	-90.17466	31
				**TOTAL**	**342**

Table S2: Correspondence between *Pocillopora* mtORF haplotypes and genomic clusters.

	Type 1a (ORF 27)	Type 2 (ORF 01)	Type 3a (ORF 46)	Type 3b (ORF 47)	Type 3d (ORF 54)	No mtORF recovered
Cluster 1	-	4	-	-	-	-
Cluster 2	10	-	-	-	-	-
Cluster 3A	31	-	-	-	-	2
Cluster 3B	126	-	-	-	-	4
Cluster 4	-	-	32	-	1	-
Unassigned	13	-	1	5	-	-

Table S3: Number of unique genotypes belonging to each *Pocillopora* genomic cluster in each region.

	Cluster 1	Cluster 2	Cluster 3A	Cluster 3B	Cluster 4	Unassigned
Bahía Culebra	-	-	1	6	5	2
Bahía Chiriquí	-	-	-	38	4	2
Clipperton	4	1	2	-	-	1
Coco	-	7	13	5	1	9
Galápagos Central	-	-	6	8	2	-
Galápagos North	-	2	11	14	-	5
Golfo Dulce	-	-	-	5	9	-
Gorgona	-	-	-	27	4	-
Panamá	-	-	-	27	8	-
**TOTAL**	**4**	**10**	**33**	**130**	**33**	**19**

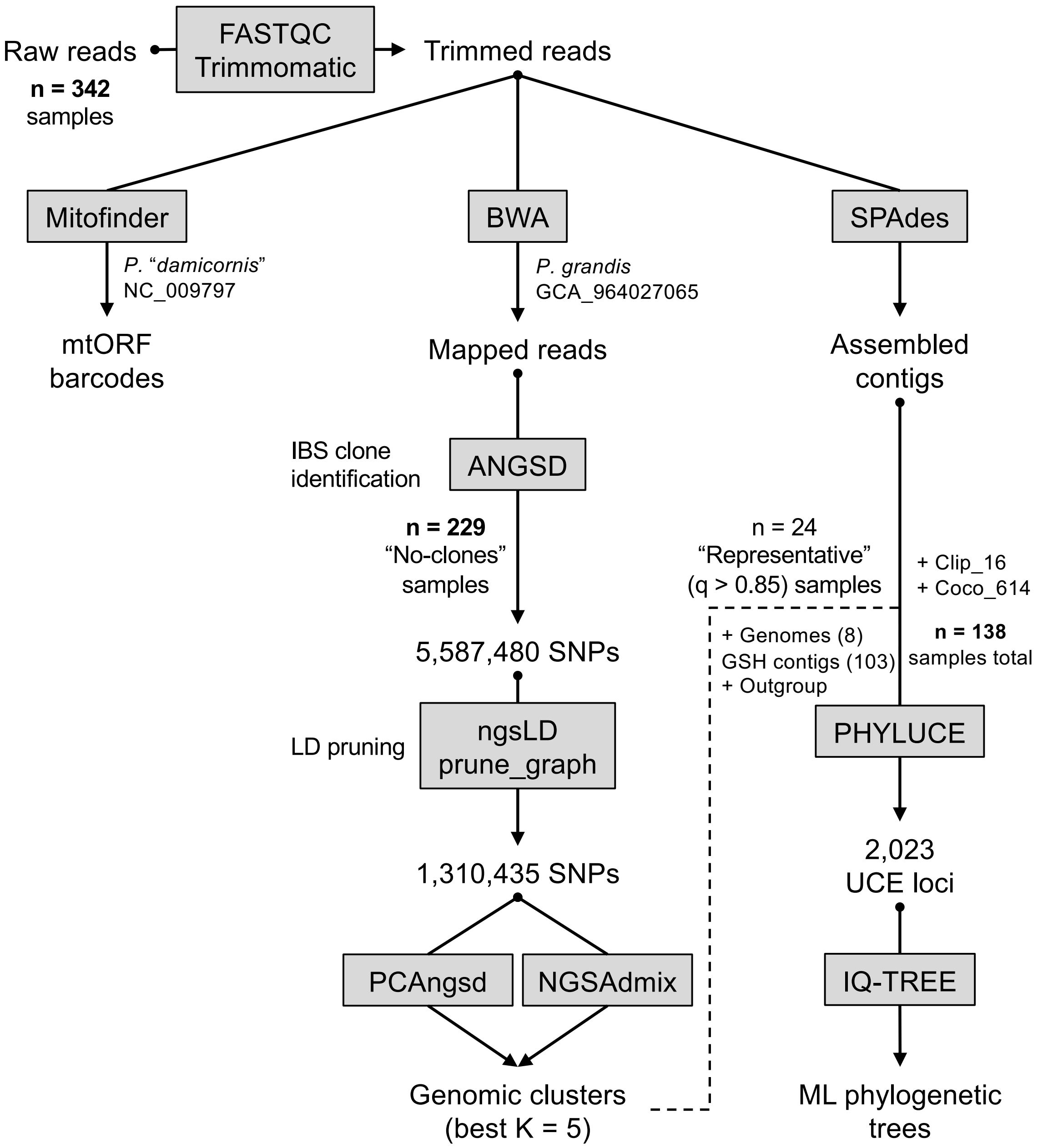

Figure S1: Genome-skimming data analysis pipeline diagram for identifying genomic clusters in ETP *Pocillopora* corals and assessing their taxonomic relationships via comparisons to genome-based species hypotheses (GSHs) proposed in Oury et al. (2023).

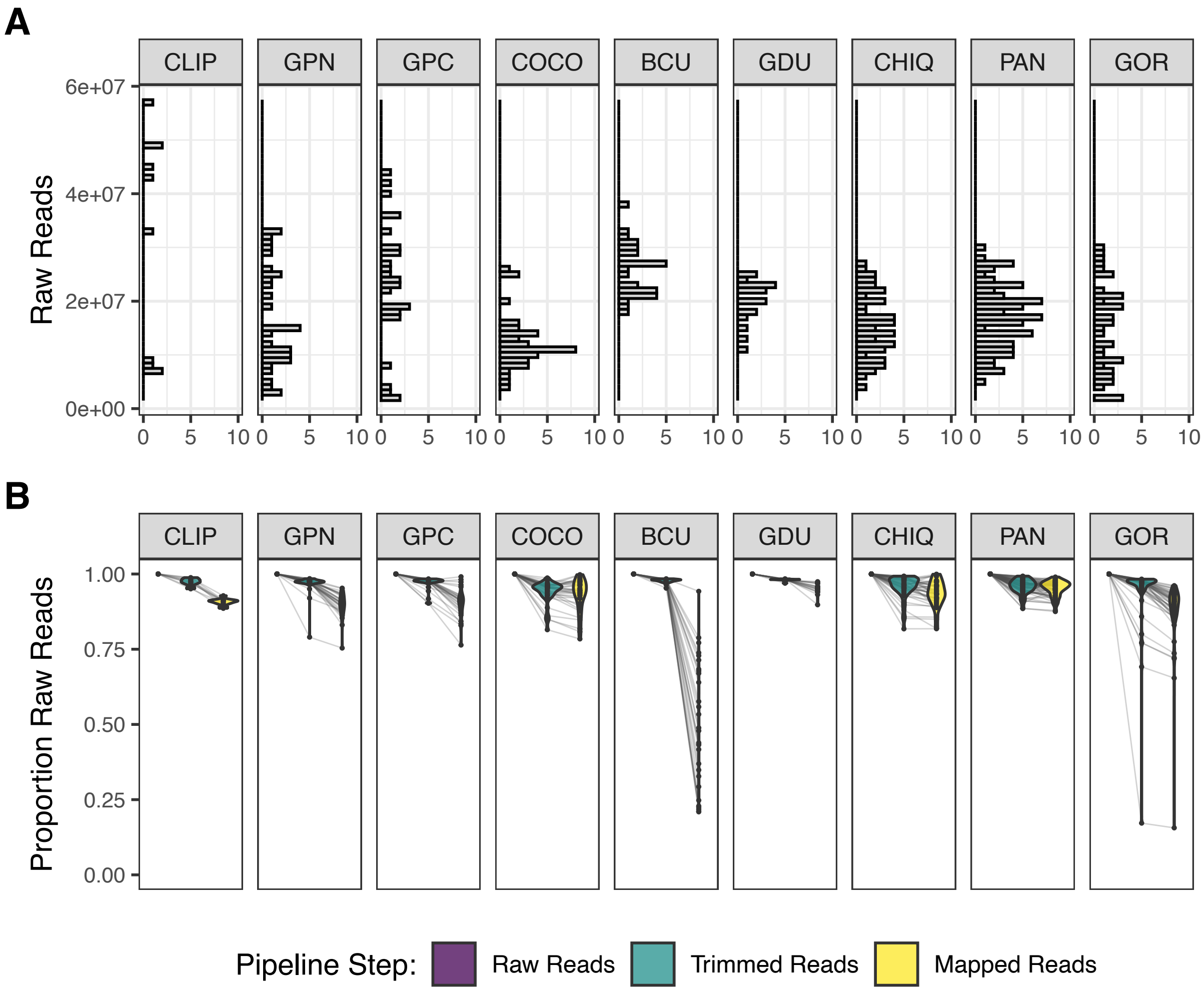

Figure S2: Genome-skimming pipeline performance summary for reference genome alignment. (A) Histograms of sample read depths by region, (B) violin plots of read proportions after trimming and mapping to *Pocillopora grandis* reference genome (GCA_964027065.2).

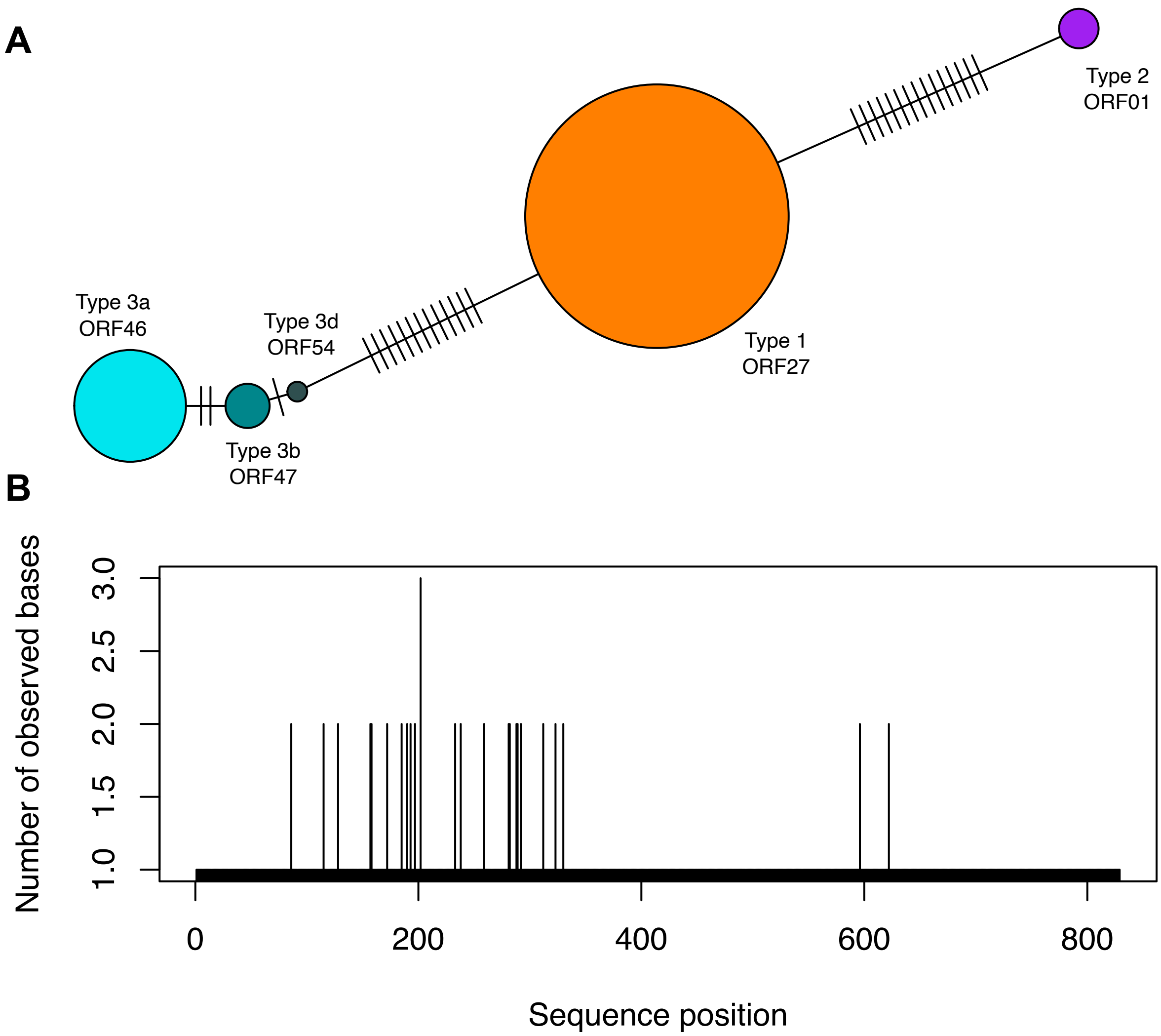

Figure S3: *Pocillopora* mtORF median-joining haplotype network and alignment summary for all recovered sequences (n = 325). (A) Haplotype network depicting the relative number of samples belonging to each mtORF type with hashes to represent the number of mutations between haplotypes, (B) number of observed nucleotide bases along the length of the 829 bp alignment.

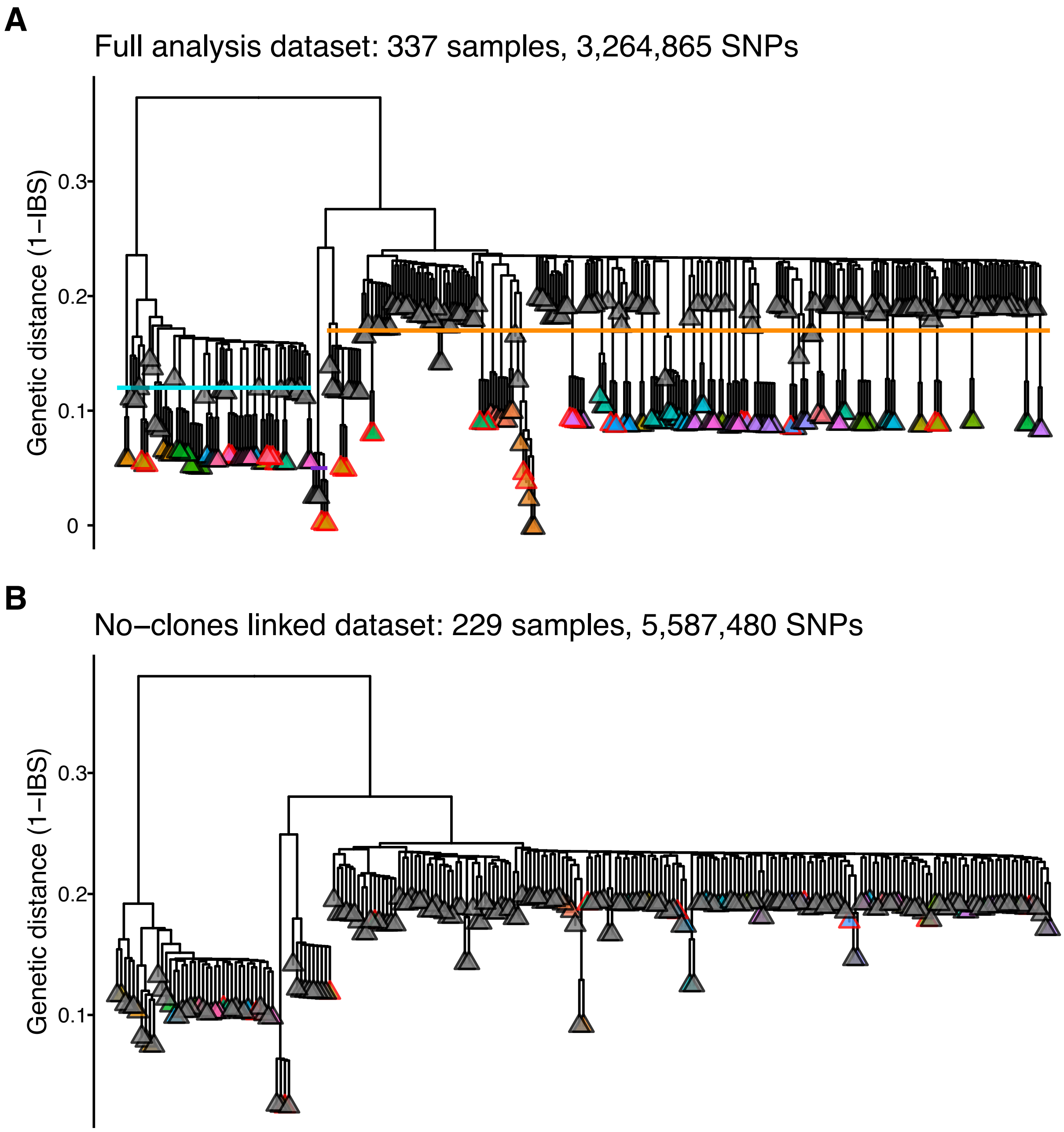

Figure S4: Identity-By-State (IBS) clustering enables *Pocillopora* clonal genotype identification. (A) IBS dendrogram for the full analysis data set (n = 337 samples, 3,264,865 SNPs). Technical replicates for each mtORF type are outlined in red, and the cut height threshold for different mtORF types is depicted as a line across the dendrogram, with orange for Type 1a at 0.17, purple for Type 2 at 0.05, and turquoise for Type 3a/3b at 0.12. Clonal groups of samples that cluster below these thresholds (and thus correspond to a single genotype) are colored according to clone group, and singleton samples (one sample per single genotype) are colored grey. (B) IBS dendrogram for the no-clones linked data set (n = 229 samples, 5,587,480 SNPs).

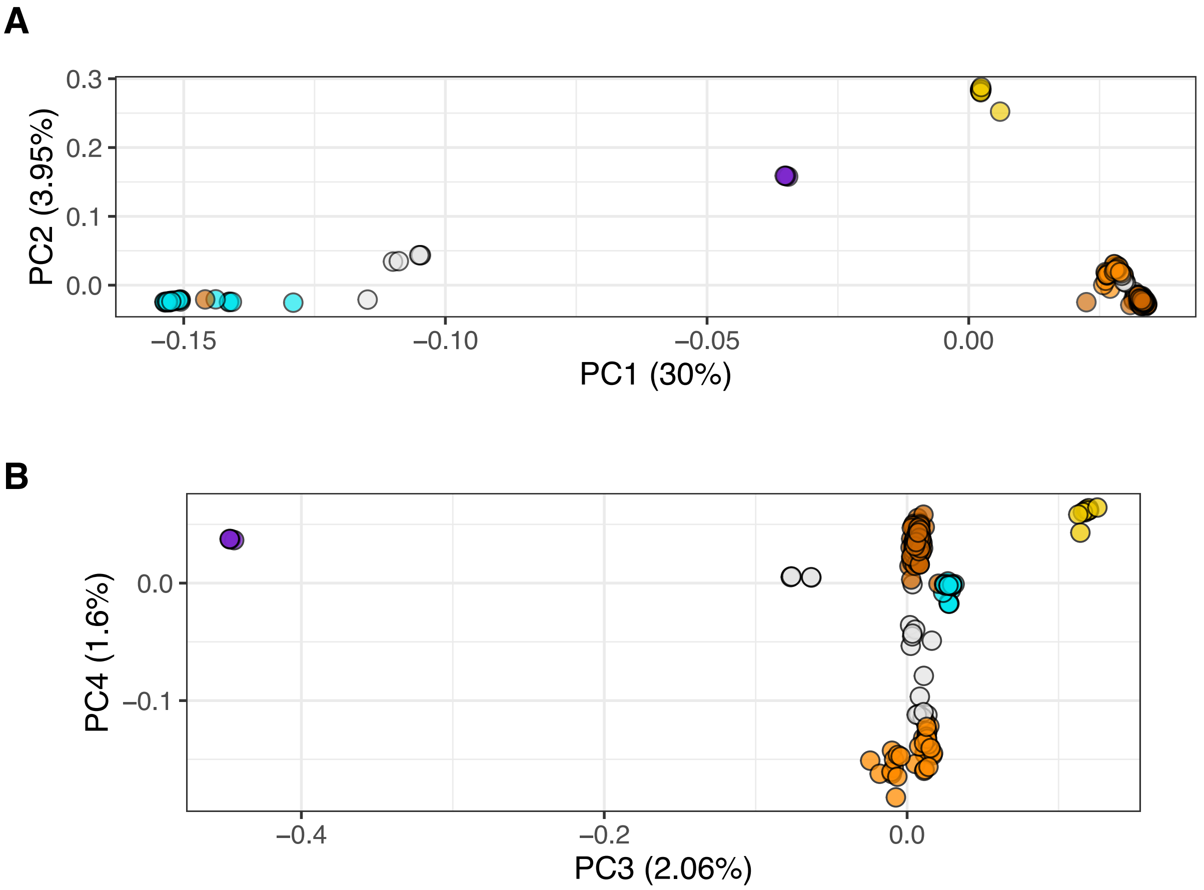

Figure S5: PCA plots generated by PCAngsd for the no-clones linked SNP dataset using (A) PCs 1 and 2 and (B) PCs 3 and 4. Clusters are colored purple for Cluster 1, gold for Cluster 2, orange for Cluster 3A and dark orange for Cluster 3B, and turquoise for Cluster 4, while unassigned samples are colored grey.

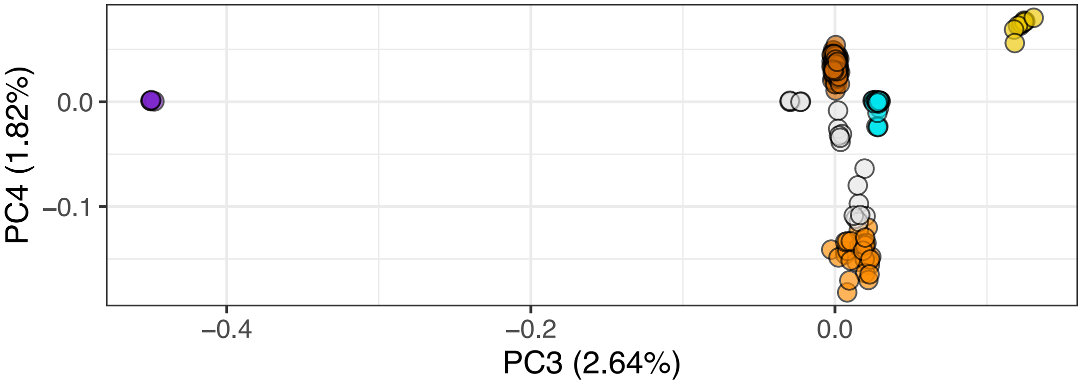

Figure S6: PCA plot generated by PCAngsd for the no-clones unlinked SNP dataset using PCs 3 and 4. PCs 1 and 2 are shown in Fig. 1B. Clusters are colored purple for Cluster 1, gold for Cluster 2, orange for Cluster 3A and dark orange for Cluster 3B, and turquoise for Cluster 4, while unassigned samples are colored grey.

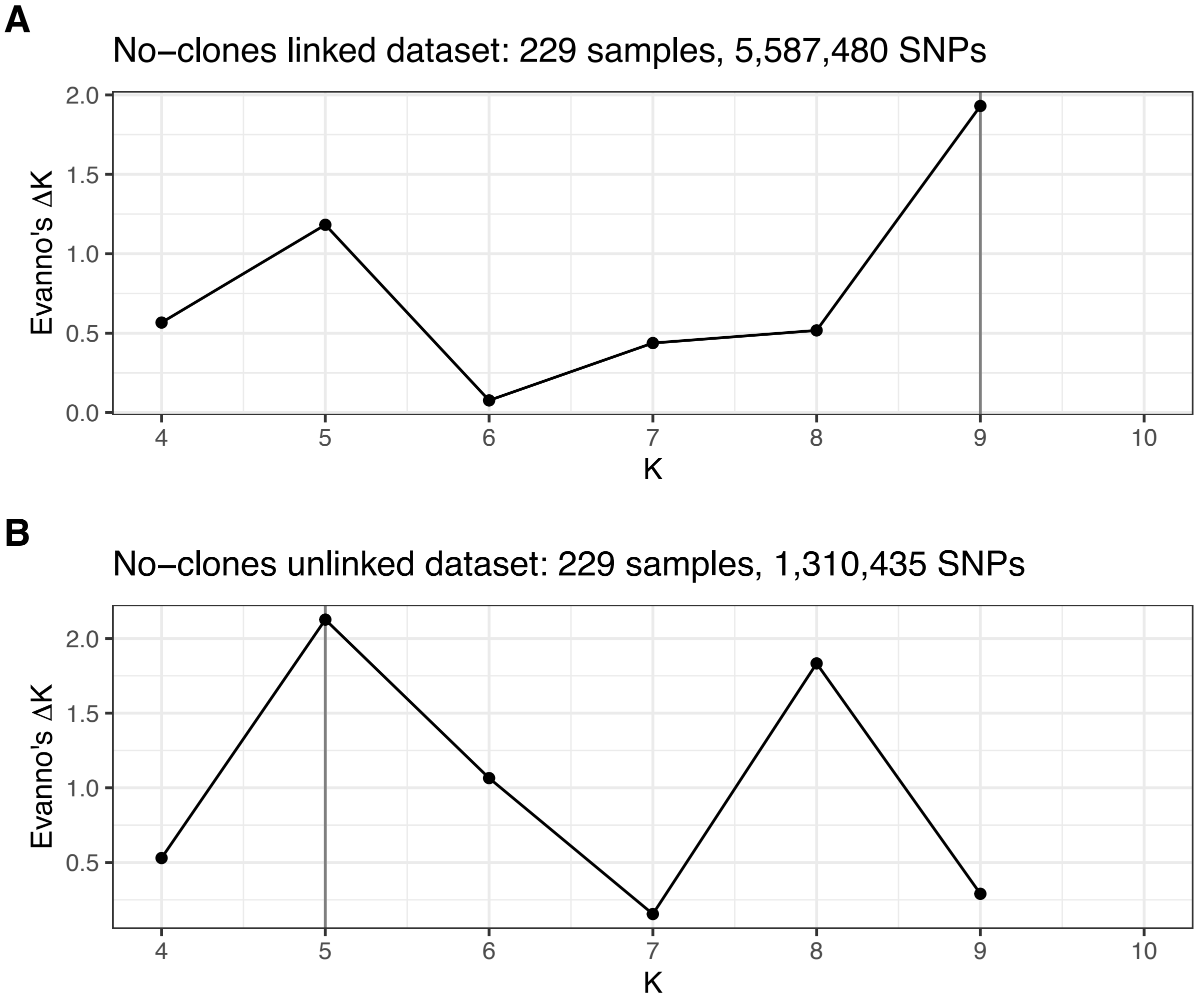

Figure S7: NGSadmix most likely K values summary. (A) Plot of the NGSadmix most likely K values using the Evanno ΔK method for the no-clones linked SNP dataset. (B) Plot of the NGSadmix most likely K values using the Evanno ΔK method for the no-clones unlinked SNP dataset. The most likely value of K = 5 was identified based on the the no-clones unlinked SNP dataset.

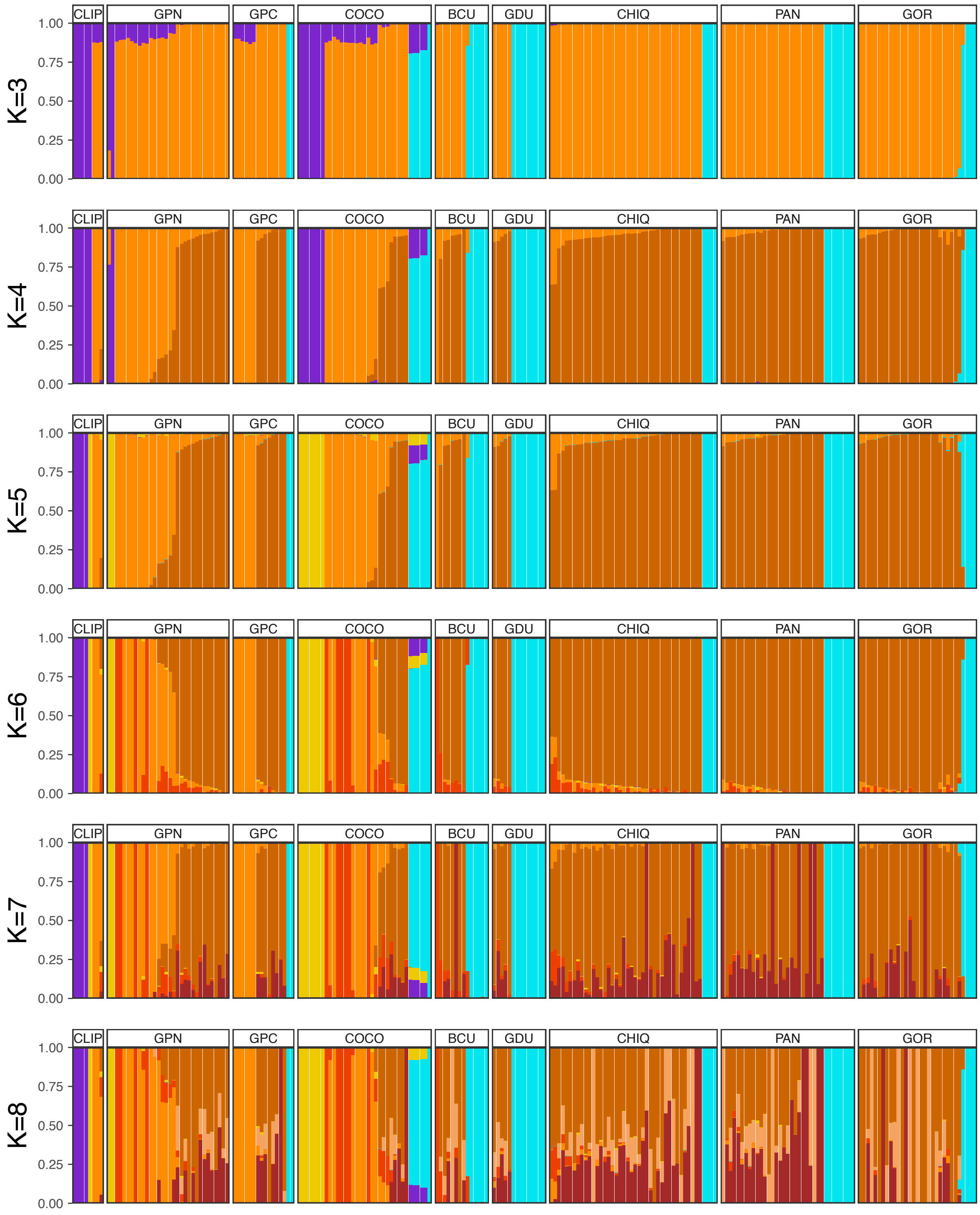

Figure S8: NGSadmix clustering results for different values of K (K = 3 – 8) based on the no-clones unlinked SNP dataset.

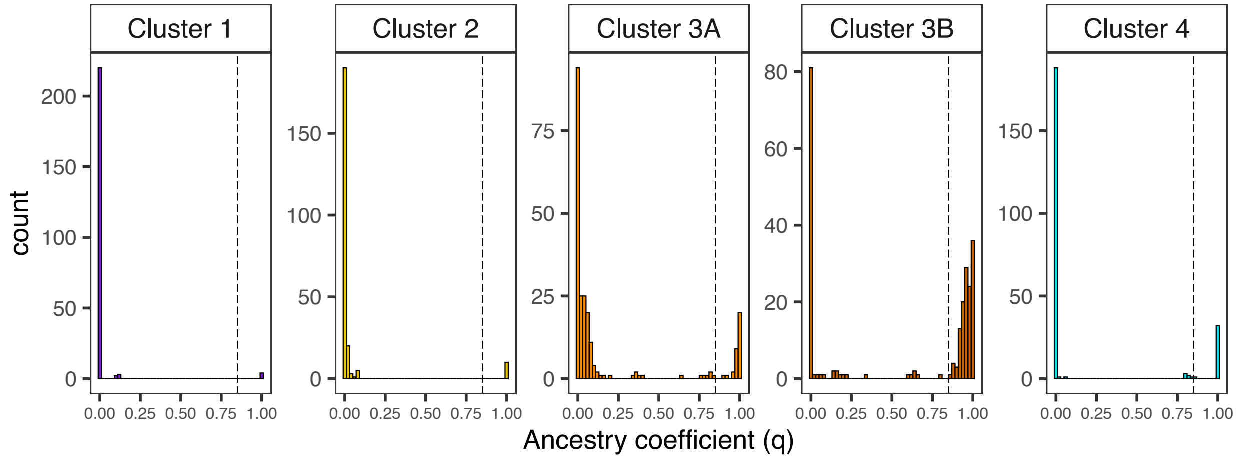

Figure S9: Histograms of ancestry coefficient (q) values for NGSadmix K = 5 clusters, depicting the number of representative samples with q > 0.85 for each *Pocillopora* genomic cluster.

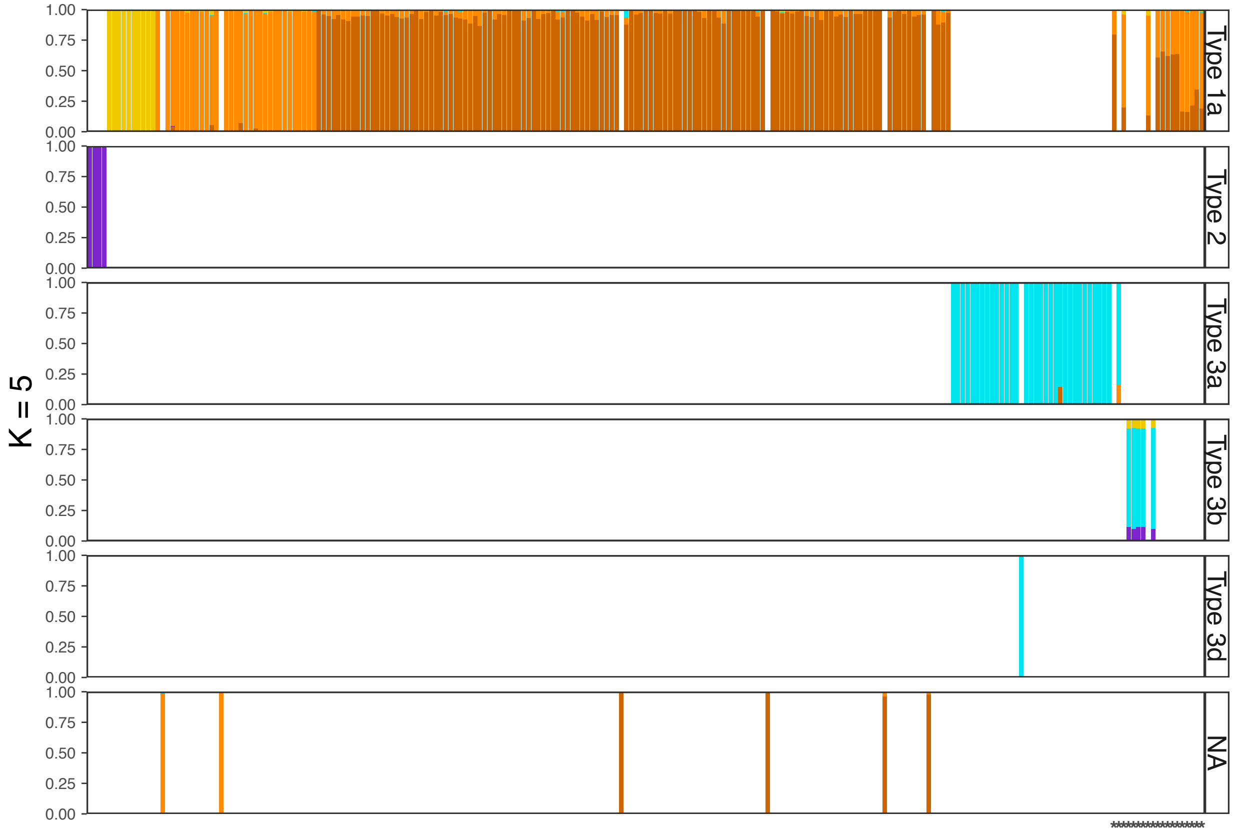

Figure S10: NGSadmix bar plots of ancestry coefficients (q) for most likely value of K = 5 facetted according to mtORF type to reveal strong correspondence between genomic cluster and mtORF type designations, unassigned samples (q < 0.85) are labelled with an asterisk.

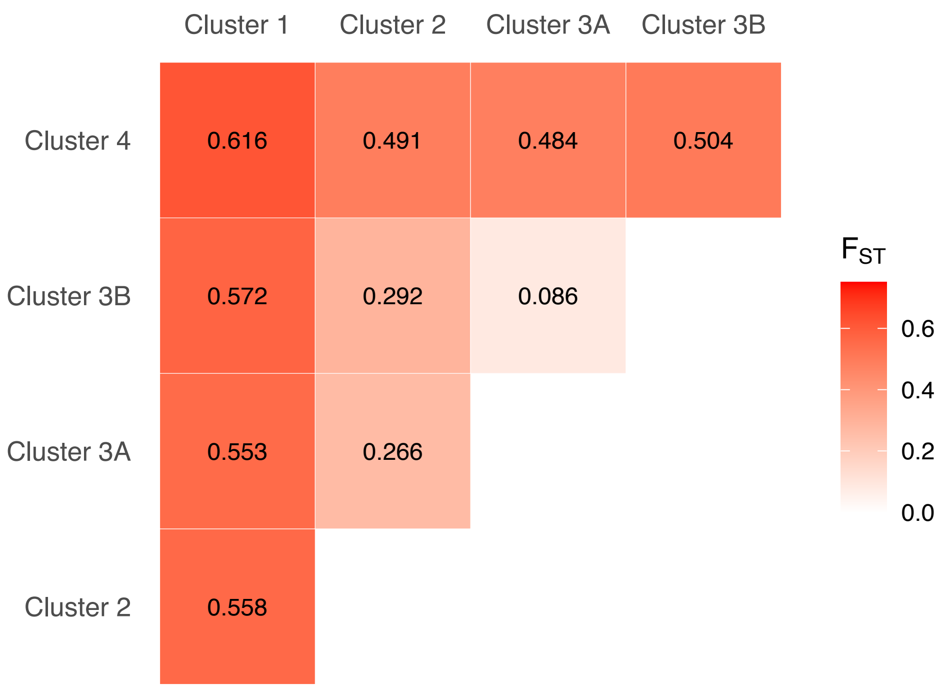

Figure S11: Heatmap of pairwise genome-wide F_{ST} between ETP *Pocillopora* genomic clusters.

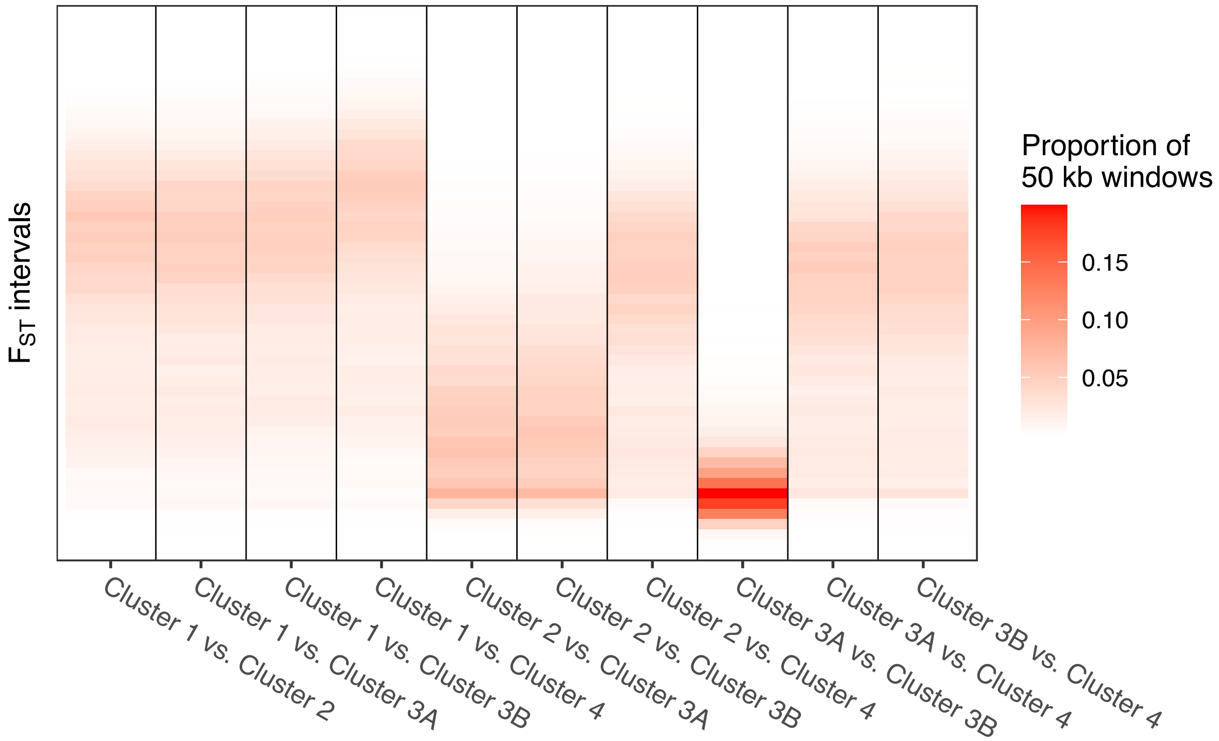

Figure S12: Heatmap of proportion of F_{ST} values in non-overlapping 50kb windows between pairs of ETP *Pocillopora* genomic clusters.

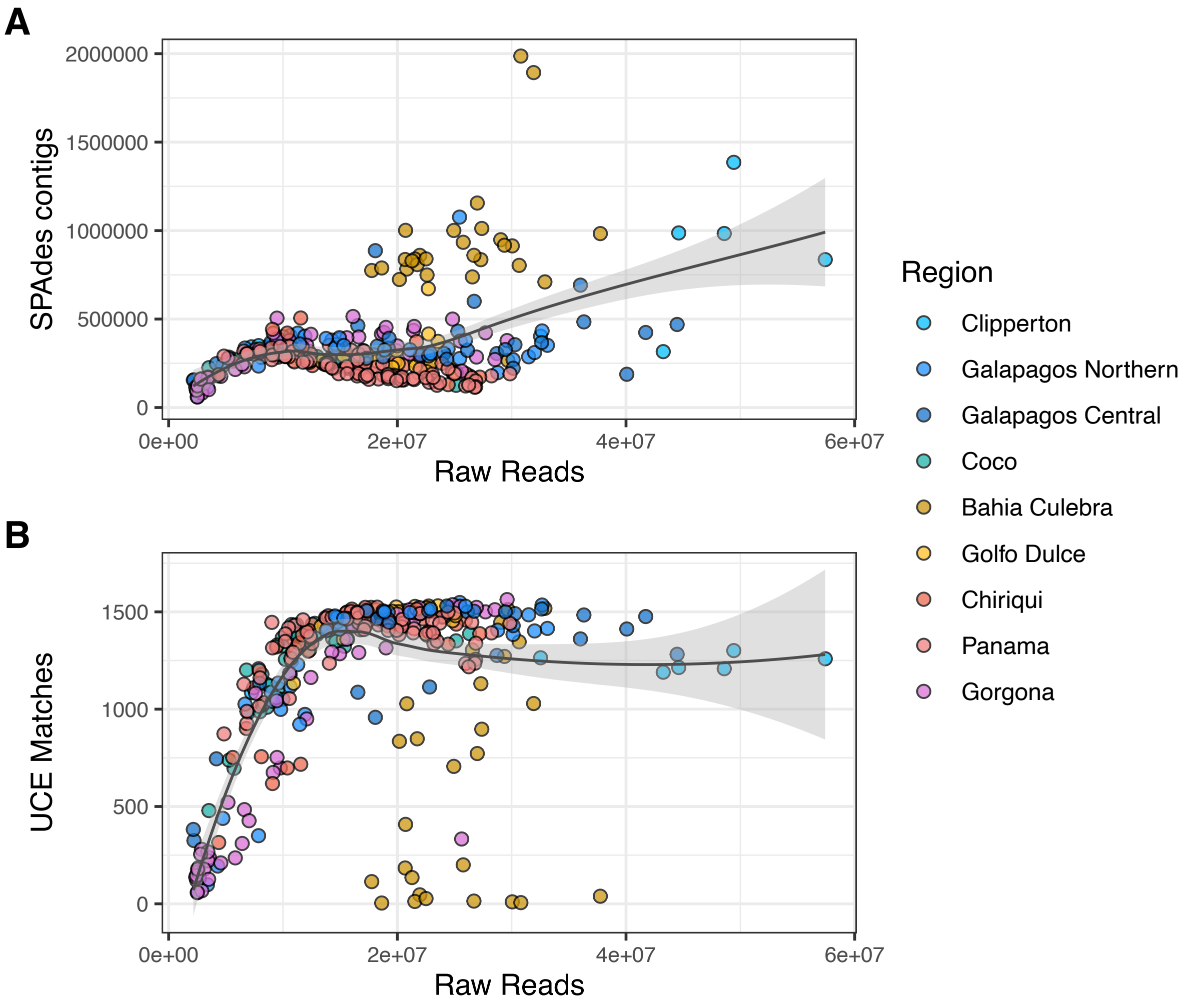

Figure S13: Genome-skimming pipeline performance summary for UCE recovery. (A) Number of assembled SPAdes contigs per sample by raw read depth, (B) UCE matches per sample by raw read depth.

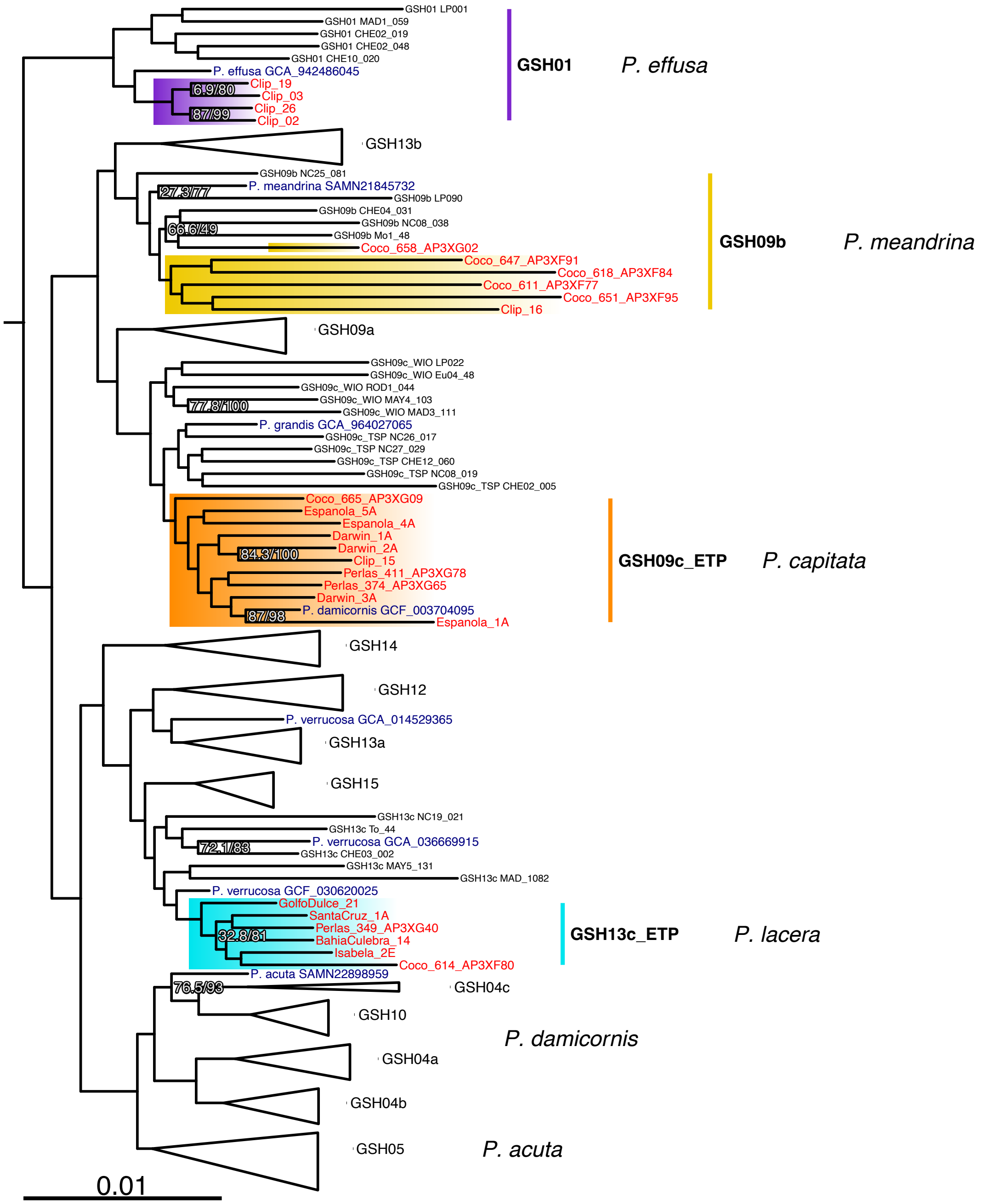

Figure S14: ML phylogenetic tree of UCE loci recovered from representative samples of ETP species-level genomic clusters and previously identified GSHs from Oury et al. (2023). The tree was constructed using a 75% data matrix with 138 taxa, 1,263 UCE loci, 613,088 bp, and 166,742 informative sites, and nodes with <90% ultrafast bootstrap (UFBoot) support are labelled with UFBoot and SH-aLRT test support values shown as UFBoot / SH-aLRT.

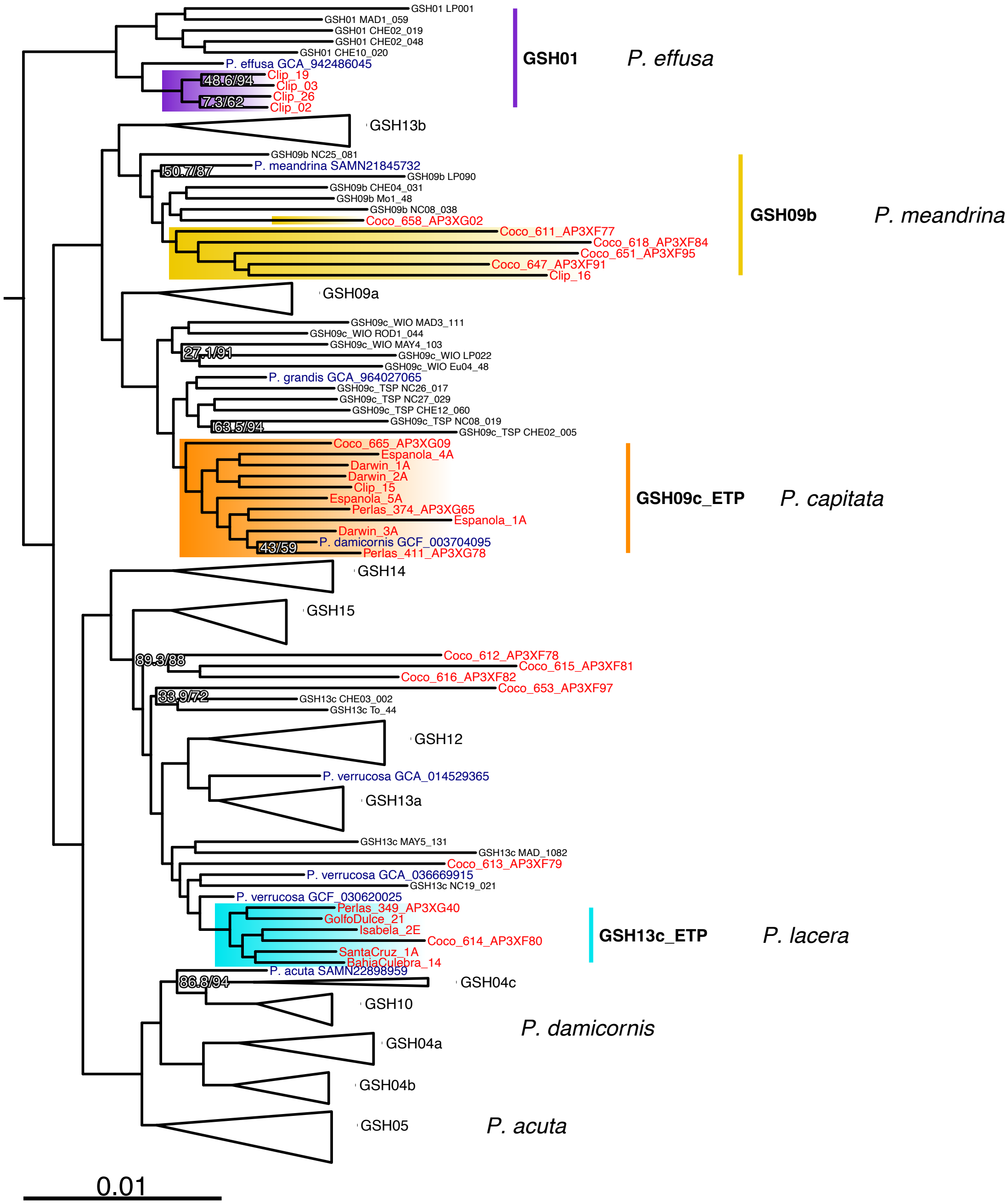

Figure S15: ML phylogenetic tree of UCE loci recovered from representative samples of ETP species-level clusters and previously identified GSHs from Oury et al. (2023), with admixed samples from Isla del Coco (all mtORF 3b samples) included. The tree was constructed using a 50% data matrix with 143 samples, 1,521 UCE loci and 710,682 bp, and nodes with <90% ultrafast bootstrap (UFBoot) support are labelled with UFBoot and SH-aLRT test support values shown as UFBoot / SH-aLRT.

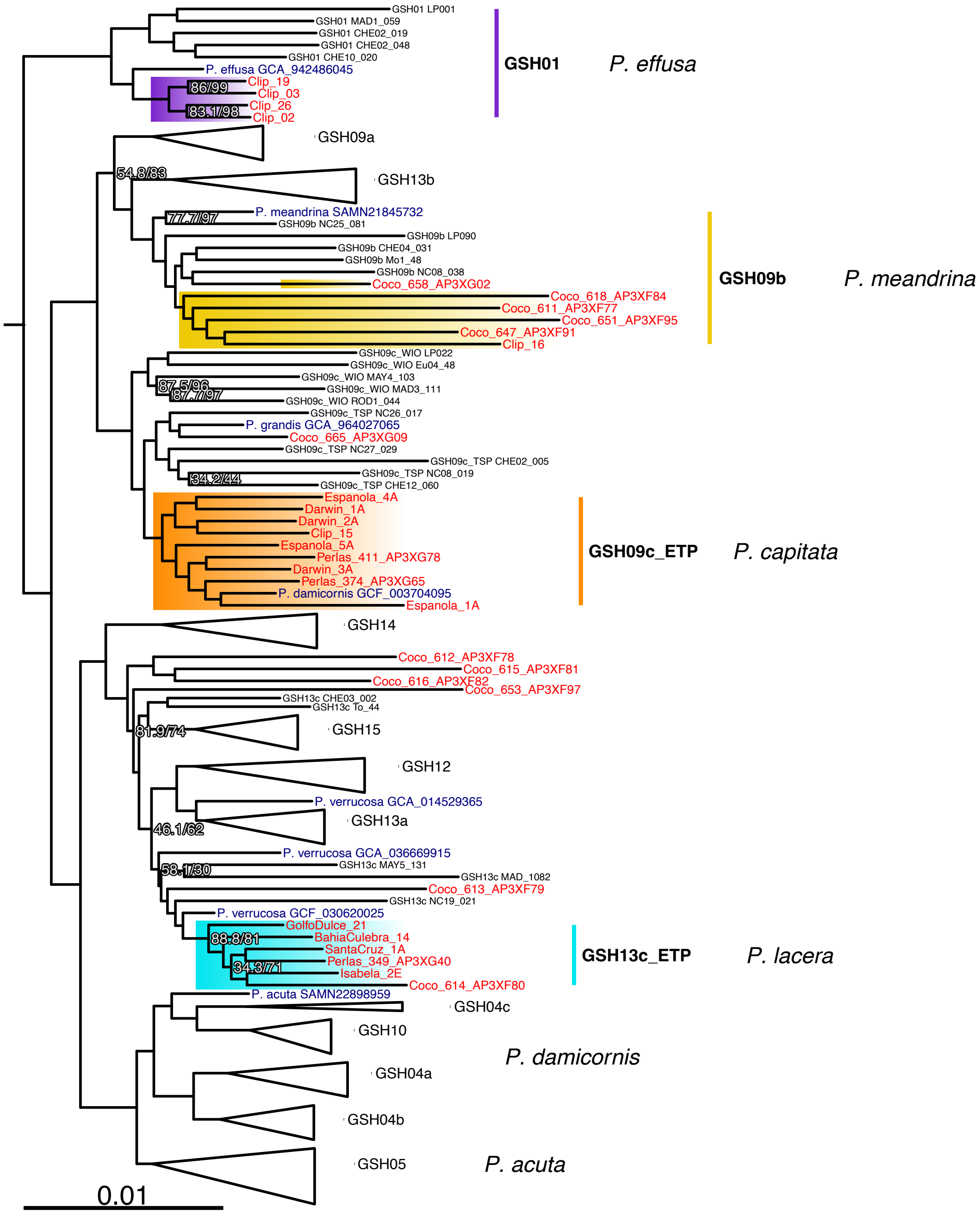

Figure S16: ML phylogenetic tree of UCE loci recovered from representative samples of ETP species-level genomic clusters and previously identified GSHs from Oury et al. (2023), with admixed samples from Isla del Coco (all mtORF 3b samples) included. The tree was constructed using a 75% data matrix with 143 taxa, 1,252 UCE loci, 599,496 bp, and 162,250 informative sites, and nodes with <90% ultrafast bootstrap (UFBoot) support are labelled with UFBoot and SH-aLRT test support values shown as UFBoot / SH-aLRT.

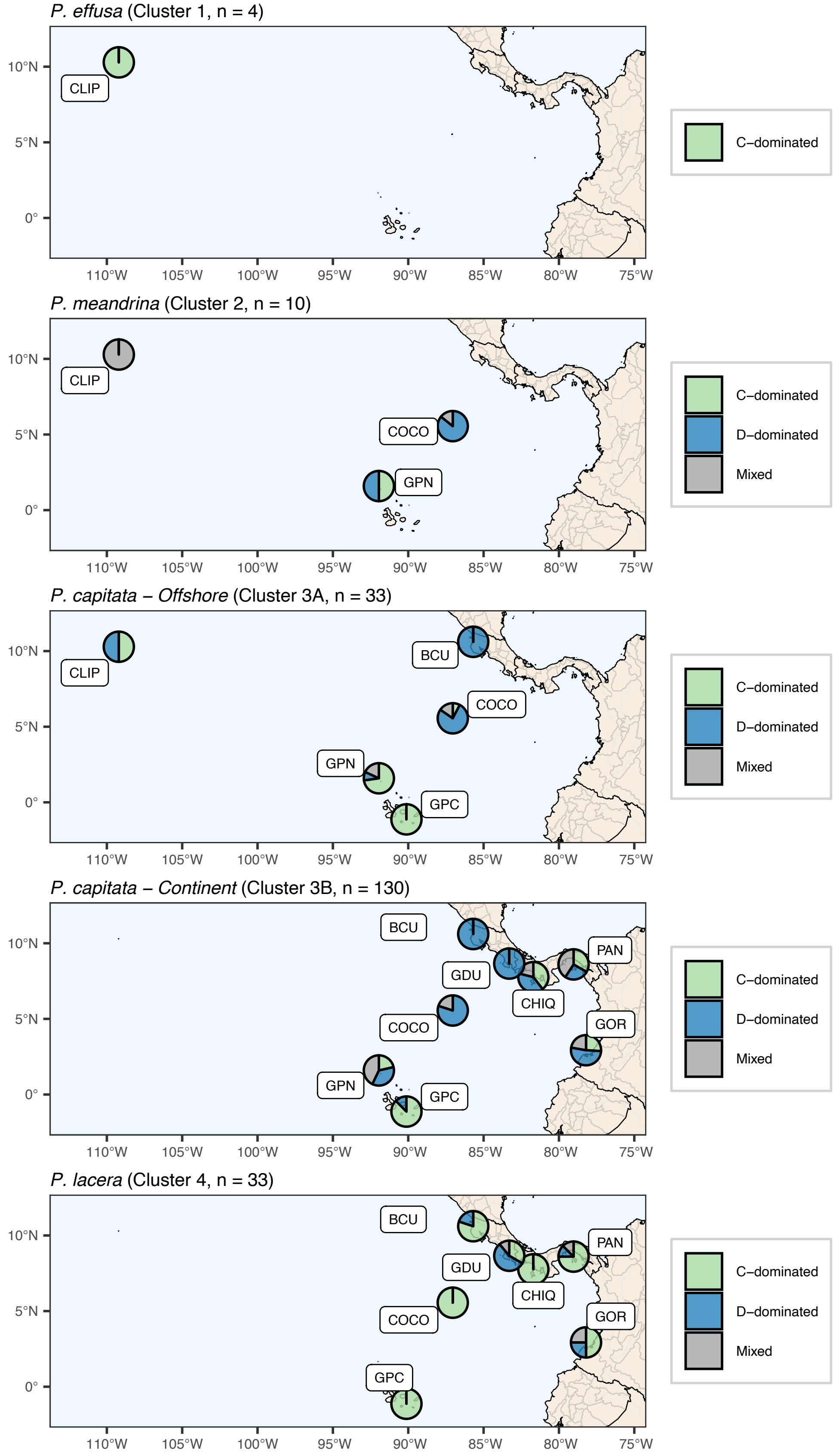
P. effusa (Cluster 1, n = 4)
CLIP
C–dominated
P. meandrina (Cluster 2, n = 10)
CLIP
COCO
GPN
C–dominated
D–dominated
Mixed
P. capitata – Offshore (Cluster 3A, n = 33)
CLIP
BCU
COCO
GPN
GPC
C–dominated
D–dominated
Mixed
P. capitata – Continent (Cluster 3B, n = 130)
BCU
GDU
CHIQ
PAN
GOR
COCO
GPN
GPC
C–dominated
D–dominated
Mixed
P. lacera (Cluster 4, n = 33)
BCU
GDU
CHIQ
PAN
GOR
COCO
GPC
C–dominated
D–dominated
Mixed
10°N
5°N
0°
110°W
105°W
100°W
95°W
90°W
85°W
80°W
75°W

Figure S17: Maps with pie charts showing the proportion of each *Pocillopora* genomic cluster's samples that are *Cladocopium*-dominated, *Durusdinium*-dominated, or mixed (<85% dominance of either genera) across sampling regions. Region codes are abbreviations for Clipperton Atoll (CLIP), Northern Galápagos (GPN), Central Galápagos (GPC), Isla del Coco (COCO), Bahía Culebra (BCU), Golfo Dulce (GDU), Gulf of Chiriquí (CHIQ), Gulf of Panamá (PAN), and Isla Gorgona (GOR).
