# Supplementary material for: Genome Skimming Illuminates Hidden Species Diversity and Symbiodiniaceae Associations in East Pacific Pocillopora Corals
Source: Genome Biol Evol. 2025 Dec 3;18(2):evaf235. doi: 10.1093/gbe/evaf235 (PMC12859748; doi:10.1093/gbe/evaf235)
Supplement: evaf235_Supplementary_Data [file evaf235_supplementary_data.zip › TaxonomicAccount_PociGSkim_111725.pdf]

TAXONOMIC ACCOUNT

Order SCLERACTINIA Bourne, 1900

Family Pocilloporidae Gray, 1840

Genus *Pocillopora* Lamarck, 1816

***Pocillopora lacera* Verrill, 1869, status revised**

Here removed from synonymy with *P. damicornis* (Linnaeus, 1758) contra Squires (1959), Reyes-Bonilla (1992)

Material Examined: YPM IZ 004490.CNB *P. lacera* lectotype; YPM IZ 004490.CNA, YPM IZ 004490.CNC *P. lacera* paralectotypes; YPM IZ 004485.CN, YPM IZ 004486.CN, YPM IZ 004487.CN, YPM IZ 004488.CN, YPM IZ 008137.CN, YPM IZ 009169.CN, YPM IZ 009170.CN, YPM IZ 009171.CN, YPM IZ 009172.CN, YPM IZ 009173.CN, **USNM 1666065***

Type locality: 'Acajutla and Pearl Islands': Reported as Acajutla, El Salvador and Las Perlas, Panamá by Verrill (1869). Lectotype is from Las Perlas, Panamá as reported in Verrill (1869).

Description of the lectotype: According to Verrill's (1869) original description, "Corallum forming irregular rounded clumps, six to eight inches in diameter and height, consisting of variously and irregularly divided, crooked branches, usually digitately lobed, or lacerately divided, at the ends. Branchlets short, irregular, often like elongated verrucae. On the larger branches the verrucae are small, irregular, and distant. Cells rather large, rounded, not crowded; sometimes with twelve to twenty-four very narrow septa, which are not abortive. Columella rudimentary or wanting. Coenchyma abundant, firm, the surface finely and evenly spinulose."

The lectotype corallum measures approximately 10 cm in length, 10 cm in width, and 13 cm in height (Fig. 1B), and is fragmented into multiple pieces. Branches are delicate, irregularly spaced, and highly ramified, and occasionally fuse at contact points (Fig. 1B). Verrucae are small, irregularly spaced, and gradually intergrade into branches. The inner corallite diameter ranges between 0.55 – 0.8 mm. Within the corallite, only short, weakly developed columella are present, with rudimentary or wholly absent septa (Fig. 1C). Short spinulae are densely and evenly distributed across the coenosteum.

Variation in paralectotypes: Corallum forms irregular rounded clumps often no more than 20 cm in diameter and height, consisting of irregular, occasionally intercalating branches 0.2-1 cm in diameter that terminate as digitately lobed or lacerate dividing tips. On larger branches there are highly irregular and unevenly spaced verrucae that typically gradually intergrade into new branches.

Remarks: The genus *Pocillopora* was described by Lamarck (1816), and the species *P. lacera* was first described by Verrill (1869) based on specimens collected by F. H. Bradley in 1866 from the Pacific coast of Panamá, including sites in Las Perlas. Vaughan (1906) also cited

^ When listing material examined, specimens sequenced in this study are highlighted in bold, and * indicates a topotype specimen, defined as a specimen collected from the type locality that is as close as possible in morphology to the original type and description.

P. lacera (Verrill 1869) as present at Tobaquilla Island, Panamá, today known as Taboguilla Island. Durham and Barnard (1952) identified several *P. lacera* specimens in the Allan Hancock Pacific Expeditions collections, documenting the species' presence in the Galápagos and Panamá provinces of the eastern tropical Pacific (ETP) and absence in the Gulf of California, and a max depth of 9 meters. However, Squires (1959) synonymized *P. lacera* with *P. damicornis* (Linnaeus, 1758) based on morphological similarity, citing the "strongly lacerate branching characteristic of this species, most verrucae being sufficiently elongated to be termed branches". Reyes-Bonilla (1992) continued this synonymy, identifying *P. damicornis* as a common and widespread species in the ETP. This work led to many studies on coral biogeography and reproductive biology in the following decades focusing on this species. Importantly, these taxonomic decisions implied that *P. damicornis* has an extremely large geographical distribution spanning the entire tropical Indo-Pacific. For example, it is recorded by Veron et al. (2016) as occurring from the eastern Pacific (Gulf of California south to the Pacific coast of South America) across the Pacific and Indian Oceans to east Africa, as far south as South Africa and as far north as the Gulf of Aqaba.

In addition, the morphological taxonomic revision of Veron & Pichon (1976), based on specimens collected from eastern Australia, used *P. damicornis* as an example of their hypothesis that many coral species exhibited considerably greater morphological plasticity than previously assumed (e.g. the influential 'ecomorph' diagram; Veron & Pichon, 1976, Fig. 58-68). This hypothesis underpinned the extensive synonymizing of nominal species by coral taxonomists in the late 20th century under the assumption that morphological variation between nominal species was simply intraspecific variation within a single, widespread species. Veron & Pichon (1976) list four nominal species as junior synonyms of *P. damicornis* (Linnaeus, 1758): *P. acuta* Lamarck, 1816, *P. brevicornis* Lamarck, 1816, *P. bulbosa* Ehrenberg, 1834, *P. caespitosa* Dana, 1846. Importantly, Veron & Pichon state that they accepted previous synonymies without further examination, therefore the decision by Squires (1959) to synonymize *P. lacera* with *P. damicornis* was maintained despite *P. lacera* not being listed as a synonym by Veron & Pichon (1976). Three other nominal species were also synonymized with *P. damicornis* by other authors following the work of Veron & Pichon (1976): *P. favosa* Ehrenberg, 1834 from the Red Sea by Sheppard (1987), *P. caespitosa* Dana, 1846 from Hawaii by Pillai & Scheer (1976), and *P. diomedeae* Vaughan, 1906 from Easter Island by Reyes-Bonilla (2002). Consequently, by the start of the 21st century a total of eight nominal species with type localities stretching from the eastern Tropical Pacific to the Red Sea were considered junior synonyms of *P. damicornis*.

More recently, Schmidt-Roach (2014) conducted the first taxonomic revision of the genus *Pocillopora* using an integrated approach combining analysis of coral skeleton morphological characters with molecular phylogenetic analysis using sequence variation in the mitochondrial open reading frame (mtORF) barcode. This work identified numerous morphological characters of the coral skeleton that were congruent with molecular phylogenetic clades. Based on this evidence, Schmidt-Roach et al. removed *P. acuta* (Lamarck, 1816), the type species for the genus, from synonymy with *P. damicornis* (Linnaeus, 1758), provided updated descriptions of other species including *P. meandrina* Dana, 1846 and *P. verrucosa* (Ellis & Solander, 1786), and described two new species: *P. bairdi* Schmidt-Roach, 2014 from the Great Barrier Reef, and *P. aliciae* Schmidt-Roach, Miller and Andreakis 2013 from subtropical eastern Australia. In

addition, Schmidt-Roach et al. (2014) used previously published mtORF data to examine the geographic distributions of lineages delineated in their study and compared them to other mtORF data from other geographic regions. Although the geographic sampling scope of the study was largely limited to specimens from Australia, this study was important because the combined use of morphological and molecular data demonstrated that 1) the taxonomic diversity of genus *Pocillopora* was greater than assumed at the time; 2) at least some of the synonymies were incorrect; and 3) that species previously considered unusual regional variants of *P. damicornis* were actually distinct species endemic to relatively small geographic regions within the Indo-Pacific (e.g. *P. aliciae*).

Meanwhile, in the ETP Pinzón (2011) documented a new mtORF haplotype, termed type 3a (GenBank HQ378760), that was distinct from the barcode for *P. damicornis* described in Australia by Schmidt-Roach (2014) and only present in colonies from the Galápagos and Panamá (Pinzón et al. 2013). However, this study did not describe this lineage as a nominal species and instead this lineage was informally grouped together with *P. verrucosa* (Ellis & Solander 1786) in Schmidt-Roach (2014).

All *P. lacera* specimens in this study were observed to possess the unique mitochondrial marker mtORF type 3a (*sensu* Pinzón et al. 2013) or ORF 46 (*sensu* Gelin et al. 2017). Studies recorded this mtORF haplotype in the western Indian Ocean (Gélin et al. 2017), however phylogenomic analysis of ultraconserved elements (UCEs) placed these samples in *Pocillopora* GSH13b, within Clade 1 (Oury et al. 2023). In this study, UCE maximum likelihood phylogenetic analysis recovered *P. lacera* as a monophyletic clade consisting solely of eastern tropical Pacific (ETP) samples nested within *Pocillopora* Clade 3 *sensu* Oury et al. (2023), distinct and separate from GSH13b samples (Main text Fig. 2).

Field appearance: Colonies appear pale brown with a pale green or yellow hue in the field, with polyp tentacles that create a “fuzzy” appearance when extended. Corallites are large and round, typically 0.5-1.5 mm in diameter and shallow, usually with 12 weakly developed septa and a rudimentary or wholly abortive columella.

Distribution: Currently recorded in the eastern tropical Pacific (ETP) from Bahia Culebra and Nicoya Peninsula, Costa Rica south to Guayas Province, Ecuador, including Galapagos Islands, Ecuador and Isla del Coco, Costa Rica, but may occur elsewhere in the ETP.

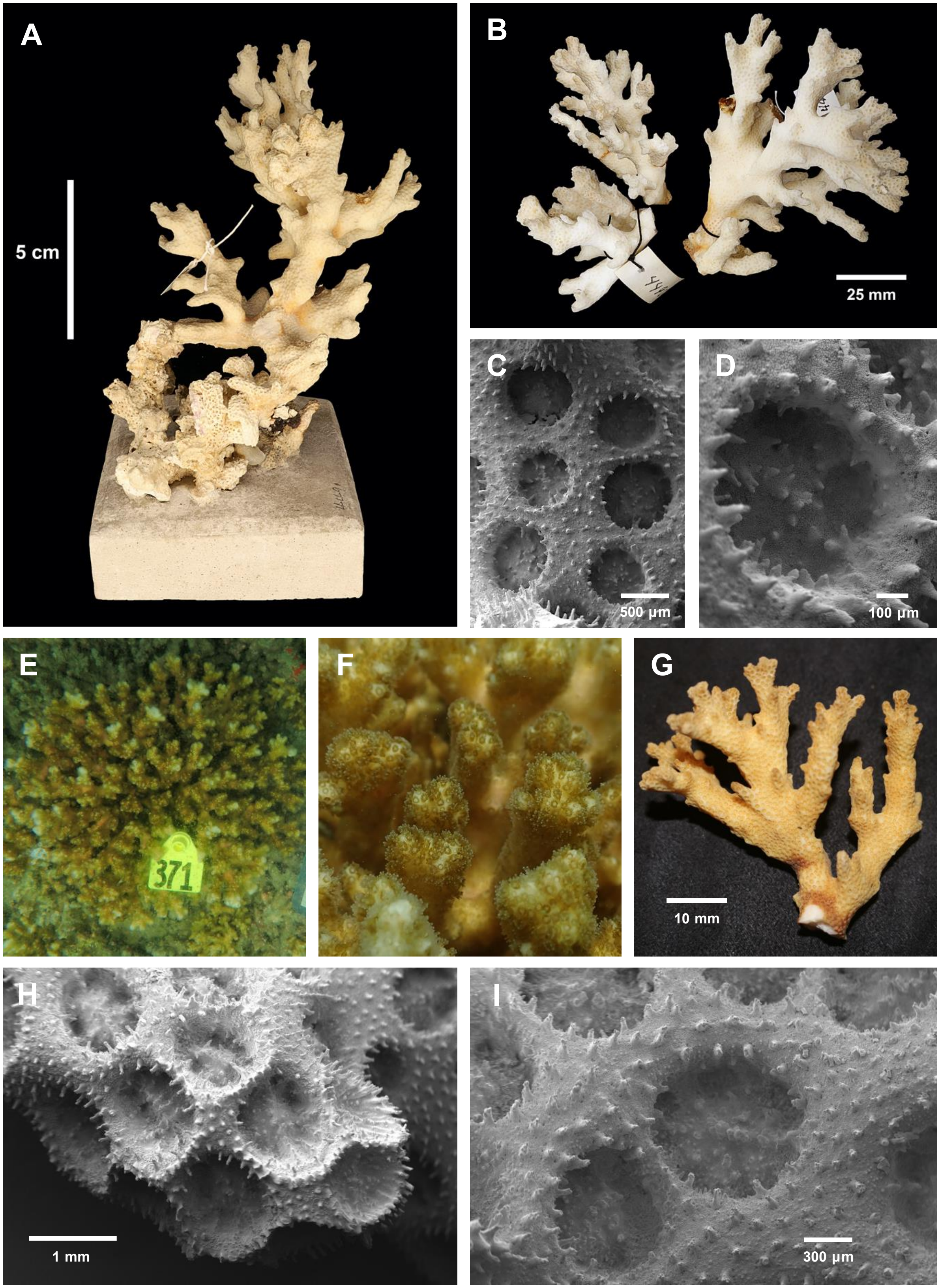
A
5 cm
B
25 mm
C
500 µm
D
100 µm
E
371
F
G
10 mm
H
1 mm
I
300 µm

Fig. 1: *Pocillopora lacera*. Skeleton fragments of type specimens (A) YPM IZ 004490.CNA *P. lacera* paralectotype, (B) YPM IZ 004490.CNB *P. lacera* lectotype, with (C) close-up of corallites and spinulae, and (D) close-up of corallite showing the weakly developed columella and septa. *In situ* appearance of topotype specimen USNM 1666065 (E) gross colony morphology and (F) close-up of branch and polyp morphology. Topotype specimen USNM 1666065 (G) skeleton morphology, (H) close-up of corallites and spinulae, and (I) close-up of corallites and weakly-developed collumella.

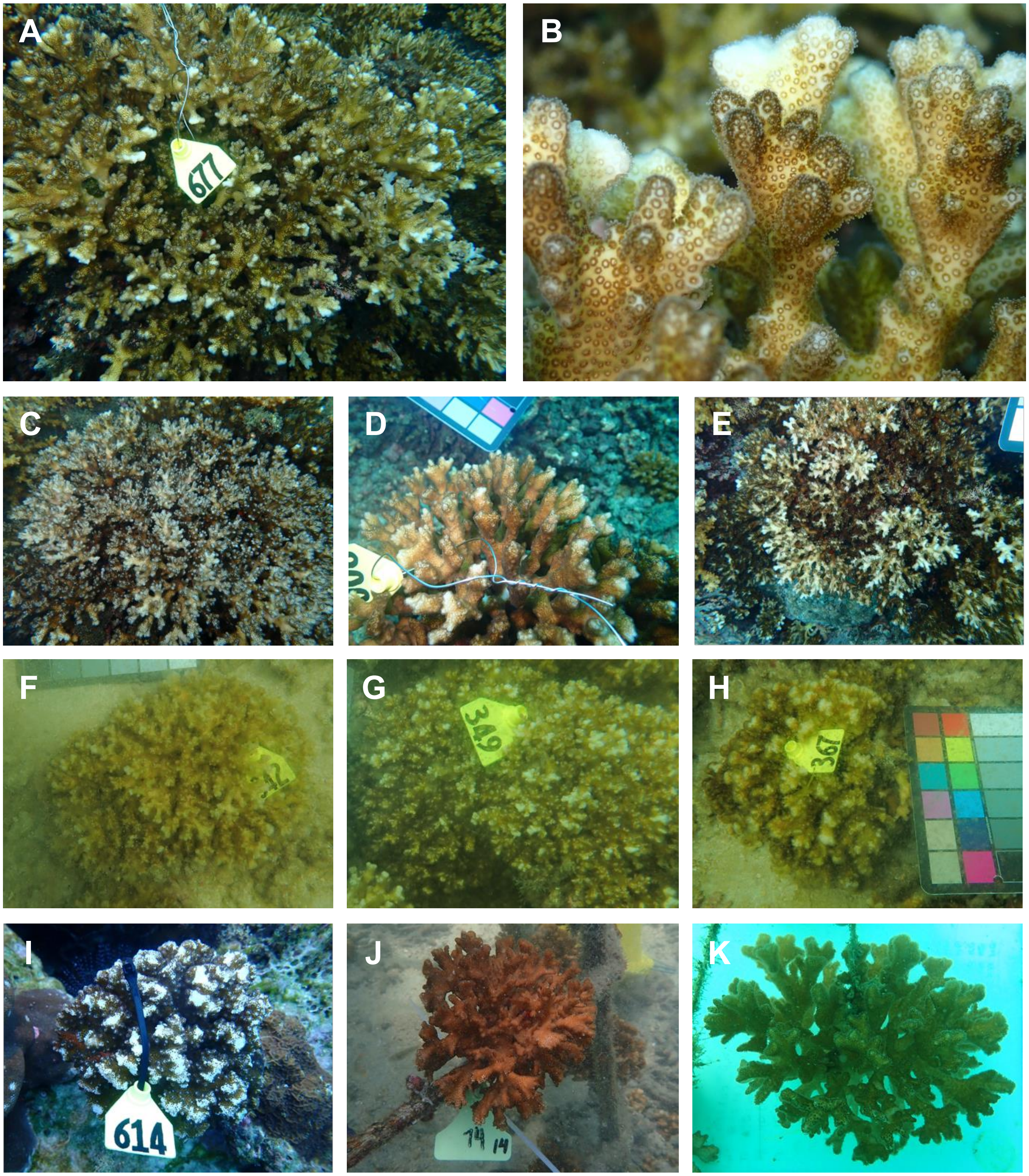

Fig 2: *In situ* photographs of *P. lacera* colonies demonstrate variation in colony morphology. Photographs of Coiba 677 (A) colony-level morphology and (B) branch and verrucae morphology demonstrate general morphological characteristics of *P. lacera* in the ETP. Additional field photographs of *P. lacera* specimens identified from field collections in (C-E) Bahia Chiriquí, Panamá, (F-H) Islas Perlas, Panamá, and (I) Isla del Coco, (J) Bahía Culebra, and (K) Golfo Dulce, Costa Rica.

References

Durham JW, Barnard J. Laurens. 1952. Stony corals of the eastern Pacific collected by the Velero III and Velero IV. Allan Hancock Pacific Exped. [Reports] 16:1–110.

Gélin P, Postaire B, Fauvelot C, Magalon H. 2017. Reevaluating species number, distribution and endemism of the coral genus *Pocillopora* Lamarck, 1816 using species delimitation methods and microsatellites. Mol. Phylogenet. Evol. 109:430–446.

Oury N, Noël C, Mona S, Aurelle D, Magalon H. 2023. From genomics to integrative species delimitation? The case study of the Indo-Pacific *Pocillopora* corals. Mol. Phylogenet. Evol. 184.

Pinzón JH, Lajeunesse TC. 2011. Species delimitation of common reef corals in the genus *Pocillopora* using nucleotide sequence phylogenies, population genetics and symbiosis ecology. Mol. Ecol. 20:311–325.

Pinzón JH, Sampayo E, Cox E, Chauka LJ, Chen CA, Voolstra CR, Lajeunesse TC. 2013. Blind to morphology: Genetics identifies several widespread ecologically common species and few endemics among Indo-Pacific cauliflower corals (*Pocillopora*, Scleractinia). J. Biogeogr. 40:1595–1608.

Reyes-Bonilla H. 1992. New records for hermatypic corals (Anthozoa: Scleractinia) in the Gulf of California, Mexico, with an historical and biogeographical discussion. J. Nat. Hist. 26:1163–1175.

Schmidt-Roach S, Miller KJ, Lundgren P, Andreakis N. 2014. With eyes wide open: A revision of species within and closely related to the *Pocillopora damicornis* species complex (Scleractinia; Pocilloporidae) using morphology and genetics. Zool. J. Linn. Soc. 170:1–33.

Squires DF. 1959. Results of the Puritan-American Museum of Natural History Expedition to Western Mexico. 7, Corals and coral reefs in the Gulf of California. Bulletin of the AMNH; v. 118, article 7.

Vaughan Thomas Wayland. 1906. Report on the scientific results of the expedition to the eastern tropical Pacific VI Madreporaria. Bull. Mus. Comp. Zool. 50:59–72.

Veron J.E.N., Stafford-Smith M.G., Turak E. and DeVantier L.M. (2016). Corals of the World. Accessed 23 Apr 2025, version 0.01. https://www.coralsoftheworld.org/page/home/?version=0.01

Verrill AE. 1869. Review of the corals and polyps of the west coast of America. Trans. Connect. Acad. Arts Sci. 6:377–558.

Verrill AE. 1869. Synopsis of the polyps and corals of the North Pacific Exploring Expedition, under Commodore C. Ringgold and Capt. John Rodgers, U.S.N., from 1853 to 1856. Collected by Dr. Wm. Stimpson, Naturalist to the Expedition. *Communications of the Essex Institute, Salem.* 6 (1): 51-104, pls. 1-2.
